# The emergence of nanoporous materials in lung cancer therapy

**DOI:** 10.1080/14686996.2022.2052181

**Published:** 2022-07-20

**Authors:** Deepika Radhakrishnan, Shan Mohanan, Goeun Choi, Jin-Ho Choy, Steffi Tiburcius, Hoang Trung Trinh, Shankar Bolan, Nikki Verrills, Pradeep Tanwar, Ajay Karakoti, Ajayan Vinu

**Affiliations:** a Global Innovative Centre for Advanced Nanomaterials, College of Engineering, Science and Environment, School of Engineering, The University of Newcastle, Callaghan, NSW, 2308, Australia; b Intelligent Nanohybrid Materials Laboratory (INML), Institute of Tissue Regeneration Engineering (ITREN), Dankook University, Cheonan 31116, Republic of Korea; c College of Science and Technology, Dankook University, Cheonan 31116, Republic of Korea; d Department of Nanobiomedical Science and BK21 PLUS NBM Global Research Center for Regenerative Medicine, Dankook University, Cheonan 31116, Korea; e Course, College of Medicine, Dankook UniversityDepartment of Pre-medical, Cheonan 31116, Korea; f Tokyo Tech World Research Hub Initiative (WRHI), Institute of Innovative Research, Tokyo Institute of Technology, Yokohama 226-8503, Japan; g School of Biomedical Sciences and Pharmacy, College of Health, Medicine and Wellness, The University of Newcastle, Callaghan, NSW, 2308, Australia

**Keywords:** Nanoporous materials, drug delivery, lung cancer, inorganic nanoporous materials, Organic nanoporous materials, stimuli responsive, hybrid nanomaterials

## Abstract

Lung cancer is one of the most common cancers, affecting more than 2.1 million people across the globe every year. A very high occurrence and mortality rate of lung cancer have prompted active research in this area with both conventional and novel forms of therapies including the use of nanomaterials based drug delivery agents. Specifically, the unique physico-chemical and biological properties of porous nanomaterials have gained significant momentum as drug delivery agents for delivering a combination of drugs or merging diagnosis with targeted therapy for cancer treatment. This review focuses on the emergence of nano-porous materials for drug delivery in lung cancer. The review analyses the currently used nanoporous materials, including inorganic, organic and hybrid porous materials for delivering drugs for various types of therapies, including chemo, radio and phototherapy. It also analyses the selected research on stimuli-responsive nanoporous materials for drug delivery in lung cancer before summarizing the various findings and projecting the future of emerging trends. This review provides a strong foundation for the current status of the research on nanoporous materials, their limitations and the potential for improving their design to overcome the unique challenges of delivering drugs for the treatment of lung cancer.

## Graphical abstract

**Figure:**
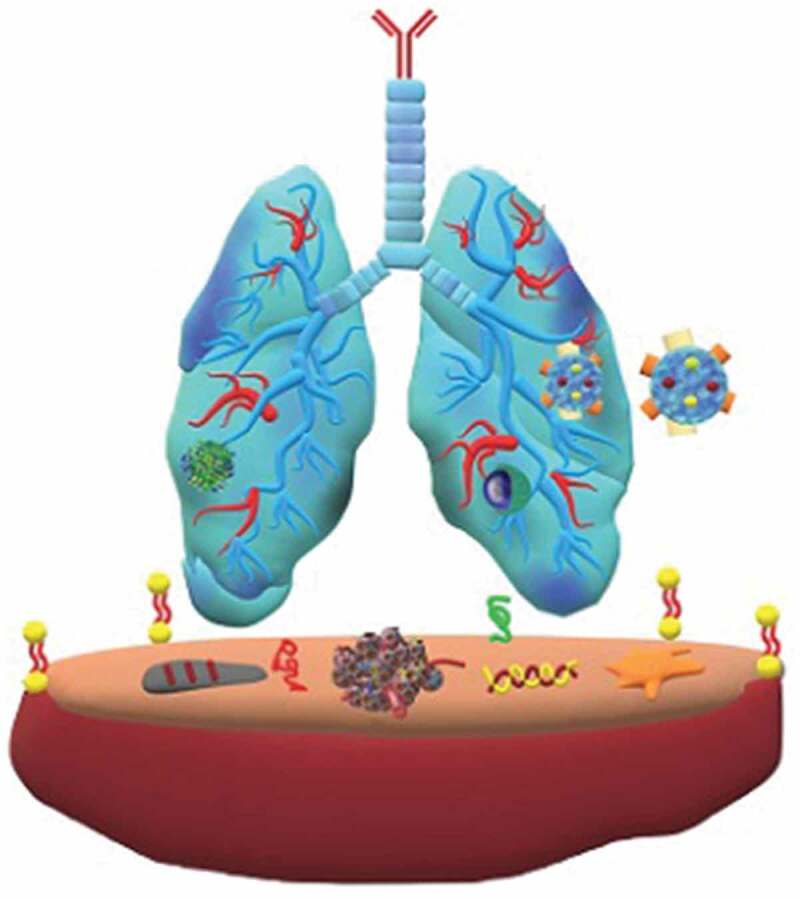

## 1. Introduction

**Scheme 1.**
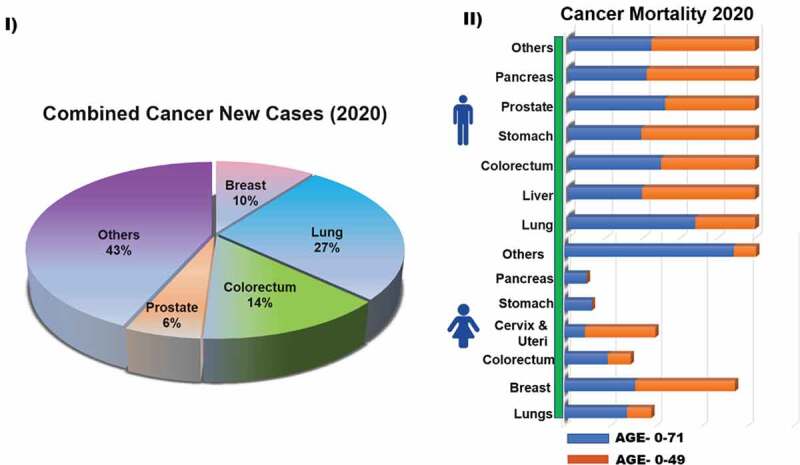
Global Cancer Statistics by WHO. I) New cancer cases in 2020 world-wide. II) Agestandardized mortality among (A) men and (B) women in 2020. For each sex, the area of the bar chart reflects the proportion of the total number of cases or deaths in each cancers from the age 0-71 and 0-49. Source: GLOBOCAN 2020 [1].

Cancer occurrence has grown significantly in the past few years to become one of the leading causes of morbidity and mortality in the human population. According to the 2020 statistics of the International Agency for Research on Cancer (World Health Organization) lung cancer has the second highest occurrence rate in both men and women (Scheme 1).

Even though previous reports have anticipated a higher incidence of lung cancer cases in men than women, last five-year reports depict a convergence of incident rates on sex differences [2]. Given that the current cancer care and treatments represent a major healthcare burden costing billions of dollars to the global economy, it is paramount to build long-term sustainable solutions for reducing cancer mortality by rapid innovation and clinical translation for the management of lung cancer [3].

Lung cancer can be categorised into two histological types i) small cell lung cancer (SCLC) and ii) non-small cell lung cancer (NSCLC). NSCLC accounts for 85% of patients and is further subdivided into three major types, adenocarcinoma, squamous cell carcinoma, and large cell carcinoma (with different subtypes-, and other types), whereas SCLC accounts for only 15% of the lung cancer types [4,5]. Like other cancers, the standard of care for lung cancers involves traditional treatments such as debulking surgery combined with radiotherapy and/or chemotherapy. In recent years, more targeted therapies, such as immunotherapy, are available to patients [6]. However, toxicities associated with the targeted therapies due to unwanted effects of drugs on healthy tissues are of significant concern and contribute to poor patient outcomes [7].

The poor clinical outcomes in lung cancer patients are attributed to (i) delayed diagnosis, (ii) concomitant diseases, (iii) lack of prophylactic modality, (iv) molecular heterogeneity of the lung cancer cells and (v) fast mutations and (vi) metastasis. The holistic management of these factors for the patients in the treatment regime is always challenging to achieve. Moreover, the delay in early-stage diagnosis and the poor drug penetration at the tumour site with the relevant therapeutic concentration are the other hurdles associated with the patient response and outcomes [8]. This has triggered significant research and development activity recently on the design of advanced therapies integrated with novel modalities, including nano-drug delivery systems. For example, the utilisation of nano-drug delivery carriers, which are designed to increase the efficacy of already existing drugs, reduce the side effects and precisely deliver the right combination of drugs to the patient for improving the patient outcomes, has made a remarkable impact on the treatment of cancer patients.

Nanosized drug delivery carriers could be composed of inorganic, organic or their hybrid materials. The advantages of nano carriers, such as, tunable size, shape, rich functionality, and the versatility in the surface modifications for efficient drug delivery in lung cancer have been demonstrated in various articles [9,10]. A quick survey of the past five-year research in this field demonstrates the pertinence of nano sized drug delivery for cancer therapies [10–17]. Even though the use of non-porous nanosized drug delivery carriers for drug/gene/peptide delivery through systemic administration in lung cancer [18,19] is popular, the research on porous nanomaterials for drug delivery in lungs have also gained a significant traction including the use of such porous nanomaterials in inhalation route of administration [20,21].

Porous nanomaterials have substantial advantages over its non-porous counterparts due to their high surface area, large pore volume, low mass density and tunable size [22]. The porous channels of the nanoporous materials can be used for capturing and delivering a large amount of poorly soluble drugs, solving one of the most significant challenges in the delivery of cancer therapeutics. Furthermore, the high surface area facilitates a large amount of drug adsorption, which potentially reduces the drug dosage cycle and number of administration to the patients. On the other hand, the diameter of the pores in nanoporous materials can be tuned to accommodate drug molecules of various sizes and shapes, from short peptides to large protein moieties [23]. The surface of the nanoporous particles can also be modified to attach several stimuli-responsive elements such as pH, temperature, redox and magnetic factors for controlling the drug release. In addition, due to the additional parameter of porosity these material can influence the aerosolization and impaction-related deposition of particles carrying the drug molecules. Aerosolization of the nanoparticles and their drug delivery has been studied since the 1990; and the size, shape and porosity of nanoparticles can affect the aerodynamics of particle deposition in lungs [24,25].

Despite the success in laboratory/experimental settings, porous nanoparticles as drug carriers are limited to few examples in clinical oncology. The new approach of using nano-porous materials for the targeted drug delivery in lungs is still an emerging concept [4]. Thus, it is important to review the recent developments about the emerging trends in the use of porous materials for drug delivery in lung cancer treatment that has shifted significantly from the discovery phase to the development phase. For example, recent trends in the drug delivery especially for cancer treatment is focused on the combination of treatments or a combination of diagnostics with treatment. This requires highly innovative approaches for the development of novel nanomaterials for carrying different types of drugs and also for delivering diagnostics with the therapy. The design of such nanomaterials includes development of unique structures, dual porosities, and innovative combination of organic and inorganic materials to form specific nanohybrid structures. It is important to critically analyse such advancements in porous nanomaterials-based drug delivery agents and identify the gaps in the research to allow the researchers to rapidly develop new structures and shorten the path from laboratory to clinical translation. Even though there are several reports on the advantages and disadvantages of natural and synthetic nanoparticles and various metal-based conjugations (gold, silver, zinc) for advanced functionalities and targeted therapies [26,27–30], a review on the opportunities of porous nanoparticles and the advantages of delivering them via inhalation and systemic routes in lung cancer has not been published yet [31,32].

Here, we have summarized the current research on nanoporous materials for drug delivery, primarily focused on treating lung cancer. The review starts by discussing the limitations of the current methods of therapy where the nanomedicine approach using porous nanomaterials can assist in improving the drug delivery outcomes. The review also outlines the selected synthesis methods and applications of inorganic, organic and hybrid conjugates of nano-porous materials as drug delivery agents. It critically analyses the role of size, shape porosity and surface charge of porous nanomaterials for their application. We have also summarized the application of nano porous materials as delivery agents treatment of lung cancer using all forms of therapy (chemotherapy, radio therapy, immunotherapy and targeted therapy) and different modes of administration. In the end, we discuss the growing research in the area of stimuli-responsive drug delivery using nanoporous materials for the treatment of lung cancer before summarizing and presenting future prospects of research in this growing field of nanoporous materials.

## 2. Current treatments and their delivery in lung cancer therapy

**Scheme 2.**
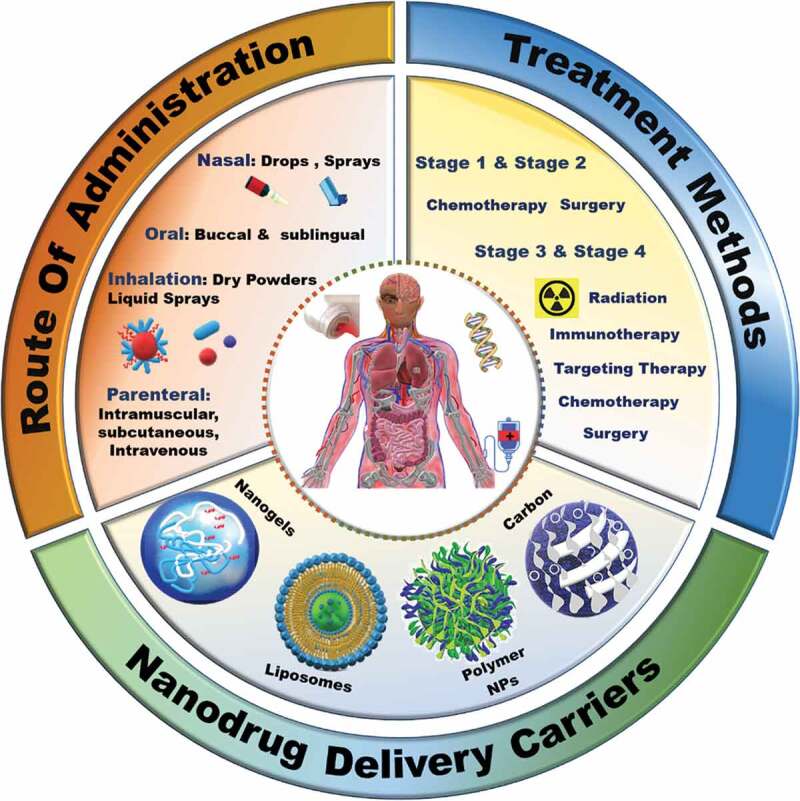
Routes of drug administration. Schematic representation of the routes of administration of anticancer drugs and the currently accepted treatment methods for various stages of lung cancer.

The lung cancer treatment methods depend on the stage of cancer (Scheme 2). Platinum-based treatments are given as baseline chemotherapy treatment for most types of lung cancers through intravenous drug administration. Several other therapies namely, radiotherapy, immunotherapy, and targeted therapy, can be combined with platinum therapy. However, these approaches can cause side effects such as nephrotoxicity, anaemia, cardiotoxicity, peripheral neuropathy, and intestinal damage [33]. Despite being used as the first line of treatment, lung cancers develop resistance against platinum-based therapy. The predicative molecular determinants of the cisplatin resistance and their gene profiling to identify the genetic variants are in progress, and it is expected that they may address the development of cancer resistance. It is reported that because of the detoxification or efficient repair of damaged DNA, the Excision Repair Cross Complementing 1 (ERCC1) gene is upregulated in cancer cells making the cells resistant to the drug [34,35]. Several other chemotherapeutic drugs like Cisplatin, Gemcitabine, Paclitaxel, Fluoropyrimidines and Pemetrexed also exhibit similar kinds of resistance in patients [35–39]. In fact, most of the drug resistance developed by cancer cells is the consequence of continuous administration of single or combinatorial chemotherapies during the treatment tenure [40–42].

Immunotherapy is another promising approach that has shown great success for lung cancer therapies, especially with fast mutating and immuno-competent lung cancer cells [43]. Treatment of lung cancers with immune checkpoint inhibitors without any genetic targeting has recently become the standard first line of treatment in many parts of the world [44]. The immune response of lung cancer can be initiated through various therapeutic vaccines, immunomodulators, monoclonal antibodies or autologous cellular therapies. MAGE-A3 targeting vaccine and liposomal-BLP25 for MUC1 peptide (mucinous carcinoma-associated glycoprotein 1) are in clinical trials for approval of stage III & stage IV NSCLC [45,46].

Similarly, Nivolumab, which targets programmed cell death receptor 1 (PD-1) on lung cancer cells, is another commonly prescribed drug to patients, and its combination with cisplatin/gemcitabine, cisplatin/pemetrexed, carboplatin/paclitaxel treatments is also under clinical trial [47]. For advanced SCLC, the vaccines such as BEC2/BCG (Bacillus Calmette-Guerin) are in clinical trials. BEC2/BCG is an anti-idiotypic antibody that mimics GD3 and are expressed on the surface of tumour cells. The study proved that administration of BEC2/BCG prolongs the survival of the cancer patients [48]. Combinatorial therapy associated with immunotherapeutic agents like ipilimumab and chemotherapeutic agents such as carboplatin and paclitaxel is also a well-known treatment, which showed an improved clinical response in various cohorts around the world [49]. However, immunotherapy suffers from several drawbacks. One of the drawbacks is that it can accrue only in solid tumours in advanced stages of lung cancers [50]. The other drawback associated with immunotherapy is the lack of tumour specific antigens that leads to off-target toxicities that affect the adrenal glands [51]. In addition, immunotherapy is also dependent on predictive biomarkers for patient selection and a lack of clinically significant biomarkers reduces the number of patients who can take the benefit of immunotherapy. Currently, most of the immunotherapies are limited to phase 2 clinical trials and were not able to achieve significant results on the cohorts in phase 3 trials [44].

Understanding the mechanism of targeted therapies to target specific oncogenic drivers in lung cancer is another crucial area of treatment. About 2/3^rd^ of lung cancer patients are reported to have a mutated gene, and among them, about half of the patients have targetable lesions [52]. Usually, most of the targeted therapies are given as an add-on treatment to baseline therapies. Several genes are identified as positive and negative markers for lung cancer. Major studies were conducted for genes like epidermal growth factor receptor (EGFR; also known as ERBB1), anaplastic lymphoma kinase (ALK), ROS1 proto-oncogene receptor tyrosine kinase (ROS1) and serine/threonine-protein kinase b-raf (BRAF) for targeted therapy in lung cancers [53–55]. However, adaptive, intrinsic, or acquired resistance is reported as a challenge for targeted therapies. As these usual therapies become redundant over time with fast mutation rates, combining two or more therapies with targeting therapy is required to achieve rapid and desired results within a short period [56,57]. Several new molecular determinants of lung cancer subtypes and their mutations are being explored, and new variants are being discovered.

These traditional and combinatorial approaches must be administered optimally to maximize the effect of the drugs and various agents. Conventionally, these drugs are loaded on a carrying agent or directly converted to an active formulation specially to increase the solubility of the drug. Recently, various materials have been explored for delivering therapeutic drugs especially for the treatment of cancers, especially lung cancer, owing to the high cytotoxicity and suboptimal tumour penetration of the current formulations [58]. A myriad of routes of administration have been used in lung cancer treatment based on the cancer stages and response of patients (represented in Scheme 2), with the most common being systemic drug administration. The systemic route generally includes parenteral, oral, transdermal, intravenous, and intramuscular routes [59]. However, issues of low sub-optimal therapeutic concentration, fast clearance of DDS by the epithelial macrophages and the corresponding toxicity of leaked drugs are major drawbacks with these routes of administration. Compared to other cancers, the administration of drugs via inhalation/pulmonary route for lung cancers is an effective modality [25]. This non-invasive route allows the desired concentration of drug to be deposited directly in the lungs without any leakage, as observed for other routes of administration, which in turn results in fast therapeutic responses [60–62]. Uniform distribution of the drugs in the alveoli, high dispersity and solubility with minimal side effects and deeper drug penetration with better patient compliance are the other advantages of pulmonary administration [63].

**Scheme 3.**
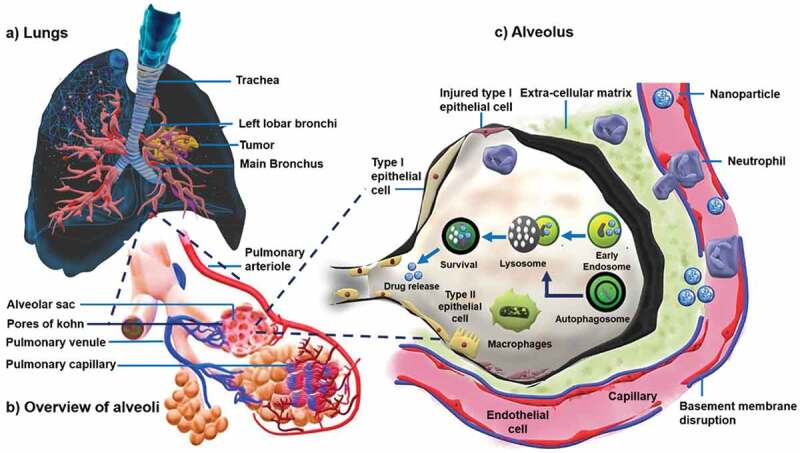
Schematic depiction of nanoparticle uptake in the alveolus. a) Lungs b) cross-section of alveolus c) cellular uptake of nanoparticles into the alveolus (adapted with permission from [68] (under CC BY 4.0).

One of the major advantages of the inhalation route is that the lungs can uptake compatible particles with larger geometric sizes of up to 10 µm with low mass density via aerosolised formulations. However, studies suggested that particle sizes which range from 1 µm to 3 µm are more respirable and can penetrate to deeper areas of the lungs [64]. The deposition of the particles in the lungs via the inhalation route is based on the forces of inertial impaction, sedimentation or Brownian diffusion [65]. The deposition and the uptake of the nanoparticle are depicted in Scheme 3. Despite promising results of the inhalation route, certain drawbacks have also been noted. The stability of the carrier containing the aerosolised drugs can be compromised, which can result in the accumulation of the particles in the upper airways or trachea. This may lead to low pharmaceutical dosage at target sites with poor distribution of drugs. By tuning the aerodynamic properties of the drugs and the nanoparticles, these shortcomings can be tackled [66,67].

Designing a better drug delivery system (DDS) that can address the challenges associated with conventional drug delivery methods which will help in translating novel therapies into clinical outcomes [69]. Among various materials, nanoparticles mediated drug delivery approaches for the treatment of cancers have gained attention in the past three decades. Several types of nanomaterials, including inorganic, organic (including polymers) and hybrid systems, have been developed to deliver the drugs directly to the cancer cells. The FDA has already approved a few nanomaterials based drug delivery systems, and numerous other nanoparticles are currently in clinical trials [70]. Several nanoparticle-based formulations containing individual or combination of drugs, either commercially approved or in clinical trials for lung cancer therapy, are listed in Table 1. It can be observed from Table 1 that the delivery medium is based on polymers or liposomes or simple formulations of polyethylene glycol (PEG).

**Table 1.** Popular therapies and their combinations that are approved or under clinical trials for the treatment of lung cancer.

Drug	Nanoparticle mediated formulations	Type of cancer	Mode of administration	Clinical trial phase	Ref
Doxorubicin (DOX)	Liposomal DOX + STM 434	Advanced solid tumours, including lung cancer	Intravenous	Phase I	[87]
	Liposomal DOX + Protein targeting L19TNFα	Advanced solid tumours, including lung cancer	Intravenous	Phase I	[88]
	Liposomal DOX + Avelumab	Metastatic or locally advanced solid tumours, including NSCLCs	Intravenous	Phase III	[89]
	Pegylated DOX + Topotecan	SCLCs	Intravenous	Phase I	[90]
	Inhaled DOX+ Intravenous docetaxel and cisplatin	Advanced NSCLCs	Inhalation+ Intravenous	Phase I	[91]
	Inhaled DOX+ cisplatin for NSCLCs	Advanced NSCLCs	Inhalation	Phase I/II	[92]
Cisplatin	Polyethylene glycol poly (glutamic acid) block copolymers and cisplatin + gemcitabine	Advanced solid tumours or squamous NSCLC	Intravenous	Phase II	[93]
	Inhaled cisplatin liposomes	Relapsed osteosarcoma metastatic to the lungs	Inhalation	Phase I/II	[94]
Docetaxel (DTX)	DTX polymeric NPs for injectable suspension	KRAS positive NSCLC and squamous cell NSCLC	Intravenous	Phase II	[95]
Paclitaxel (PTX)	NAB- PTX	Advanced NSCLC	Intravenous	FDA approved on 12^th^ October 2012	[96]
		Stage IV NSCLC			
		NSCLC with EGFR mutations			
	Polyethylene glycol 5000-distearoylphosphatidylethanolamine loaded paclitaxel	Clinical trials with patients NSCLC	Inhalation	Phase II	[97]
Paclitaxel combination	NAB- PTX + Carboplatin and HLX10	Stage IIIB/IIIC or Stage IV squamous NSCLC	Intravenous	Phase III	[98]
	NAB- PTX + carboplatin and pembrolizumab (MK‐3475, KEYTRUDA®)	Metastatic squamous NSCLC	Intravenous	Phase III	[99]
	NAB-PTX gemcitabine, cisplatin and recombinant human receptor tyrosine kinase ephrin type‐B receptor 4 (EphB4)‐HSA fusion protein	Advanced or metastatic solid tumours	Intravenous	Phase I	[100]
	Paclitaxel liposome+ Nedaplatin	Advanced Squamous NSCLC	Intravenous	Phase II	[101]
	NAB-PTX + Nintedanib.	Relapsed NSCLC adenocarcinoma	Intravenous	Phase I/II	[102]
	NAB-PTX + Atezolizumab and carboplatin	NSCLC	Intravenous	Phase II	[103]
Camptothecin	Camptothecin conjugated to a linear, cyclodextrin‐polyethylene glycol + Olaparib	Lung neoplasms and SCLC	Intravenous	Phase I/II	[104]
	Aerosolized liposomal Camptothecin	Metastatic or recurrent cancer of the lung	Inhalation	Phase I	[104]
Topotecan	Topotecan liposome	Advanced solid tumours including lung cancer	Intravenous	Phase I	[105]
Losartan and telmisartan	Polystyrene nanoparticles loaded drug	Small cell lung cancer	Inhalation		[106]

Recently, the focus of nanomaterials-based drug delivery systems is shifting towards the use of porous nanomaterials due to their several advantages over the non-porous nanoparticles. Most specifically, porous materials have a higher surface area as compared to the non-porous particles that allows higher loading of drugs per unit volume of the material of the same size. Similarly, the size of the pores can be tuned to accommodate drugs of different sizes as compared to non-porous materials that are purely dependent on surface adsorption. They also have higher sedimentation potential and better dispersity in the lungs and can be tuned to give more physical and chemical stability [71,72]. The possibility of creating core-shell structures and two different sizes of pores within the same material also makes porous material more versatile as compared to non-porous materials. Also, the porous networks help in adsorbing poorly soluble drugs and improve their delivery and distribution [73]. The release profile of porous materials can also be tuned in a predictive and reproducible way to allow controlled drug delivery. For example, the pores can be blocked (after drug loading) with agents that dissolve gradually or only upon receiving specific stimuli, allowing better control of the drug release properties. These properties make them attractive candidates for the drug delivery systems for cancer treatment. This has been witnessed recently by the growing body of literature on the development of novel porous materials, including organic, inorganic, and polymeric materials for drug delivery applications.

## 3. Synthetic routes to porous nanoparticles and their structure–function relationship in drug delivery

According to the IUPAC, porous materials can be classified into three different groups based on their pore size viz. microporous (pore size less than 2 nm), mesoporous (pore size between 2 nm and 50 nm) and macroporous (pore size greater than 50 nm) [74,75]. The microporous materials are used to adsorb small molecules such as small chemicals, peptides or amino acids, while the mesoporous materials can be used for immobilizing any moieties in between the size of 2–50 nm that includes several chemotherapeutic drugs, immuno drugs, therapeutic proteins and genes [76,77]. Materials with large sized mesopores and macropores can be used for adsorption and the delivery of proteins, peptides, genes or vaccines [78–86].

### 3.1 Synthesis of nanoporous materials

**Scheme 4.**
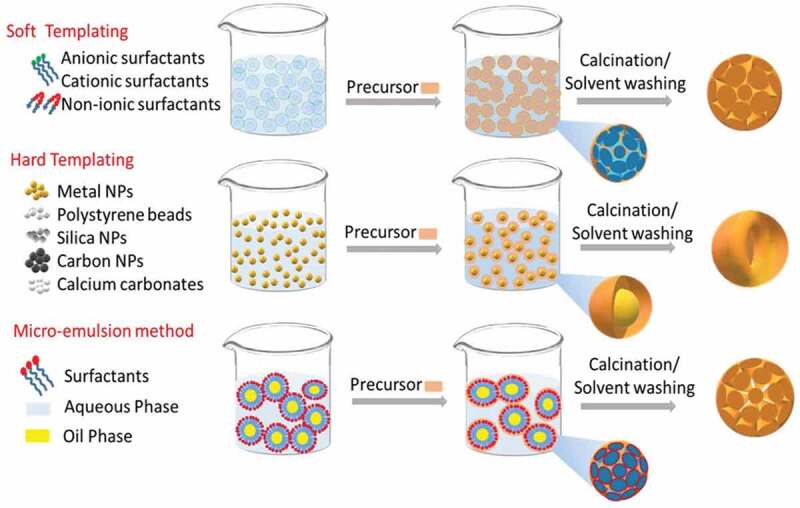
Synthesis of nanoporous materials. Schematic depiction of different approaches for the synthesis of nanoporous inorganic materials.

Drug delivery using nanoporous materials requires the synthesis of monodispersed particles that are stable and well suspended in serum. The pores for storing the drugs should be in the size range required for the encapsulation of drugs, and an orderly arrangement of pores with good accessibility helps to load a higher amount of drugs within the pores. However, designing such monodisperse nanoporous materials with an ordered arrangement of pores requires specific chemicals and involves unique synthetic processes, including chemical sol-gel process, hydrothermal, precipitation and auto-combustion [107,108]. A full description of all the synthesis processes is outside the scope of this review and has been covered elsewhere in thorough reviews about the synthesis of porous materials [109]. In this review, we present a few selected methods for the synthesis of porous inorganic materials below and the various synthetic processes involved are depicted in Scheme 4.

#### 3.1.1 Sol-gel method and soft templating approaches

The sol-gel process is the most common method for the synthesis of nanoporous materials [110]. In 1968, Stöber et al. synthesised silica nanoparticles by hydrolysing tetraethyl orthosilicate in an alkaline solution of water and alcohol through the following reaction mechanism [111].

Si(OEt)_4 _+ 4H_2_O → SiO_2 _+ 2H_2_O + 4EtOH

**Figure 1.**
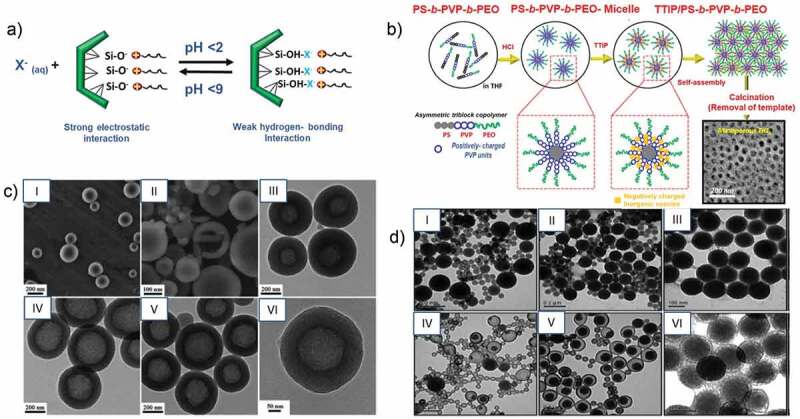
Schematic illustration of soft templating approaches and microscopic images of porous nanoparticles. **A**) Effects of pH value on the silica condensation rate. B) Schematic preparation techniques of Pt-decorated HMSN by ‘polymeric micelle assembly. C) SEM images of HMSN (I) Dispersed HMSN (II) Crushed HMSN (III, IV, V) HMSN prepared with different copolymers PS35-b-PAA4, PS58-b-PAA4 and PS113-b-PAA4, respectively. (VI) TEM images of HMSN. D) TEM images of hollow core and yolk-shell silica nanoparticles by microemulsion with different amounts of APTS ethanolic solution: (I) 50 mL (III) 100 mL (VI) 200 mL and after soaking in H_2_O for a week (II) 50 mL (IV) 100 mL (V) 200 mL (Reproduced with permission from 1A [114]. (Copyright 2002) Acc. Chem. Res, American Chemical Society 1B [115]. (Copyright 2014) Langmuir, American Chemical Society, 1C [116]. (Copyright 2014) Dalton Transactions, Royal Society of Chemistry, 1D [117]. (Copyright 2009) Chemical Communications, Royal Society of Chemistry).

The Stöber method has since been further modified to prepare various types of porous silica nanoparticles with a slight modification of the synthesis process, which involves the addition of the template. In the sol-gel process, initially, a homogeneous solution of a template in water or aqueous-solvent mixture is prepared, followed by the addition of an inorganic precursor that undergoes hydrolysis in an acidic or alkaline environment to form ionic precursors. These ionic precursors deposit on the templates transforming to a sol, and slowly forms a gel over a period based on the amount of catalyst and the aging conditions [112]. The condensation and solidification result in the precipitation of the final product. Pores are created by the removal of the template through simple calcination or solvent washing, and this approach is called the soft-templating approach. Micelles or supramolecular aggregates of surfactants are generally used as soft templates [109,113]. These pore creating molecules are called structure-directing agents (SDA) or porogens. These SDA include cationic surfactants (alkyl trimethyl quaternary ammonium surfactants such as CTAB (cetyltrimethylammonium bromide), gemini surfactants, bolaform surfactants) anionic surfactants (sodium lauryl sulfates, sodium dodecyl sulfates) and non-ionic surfactants (pluronics, triton, tween and spans) (Figure 1A) [109,114].

The interaction between the SDA and the silica precursors and the charge of the SDA in the solvent system can induce the self-assembly of the SDA (Figure 1B) into stable shapes (spherical or cylindrical micelles) [115,118,119]. The size and shape of the micelles, which eventually dictate the final morphology and structure of the materials, may be tuned by varying the solution pH, the temperature, pressure, type of solvents, the concentration of the SDA or the inorganic precursors and the rate of stirring [109,120]. The pore size of the silica nanoparticles can also be controlled by varying the hydrophobic chain length of the SDA or the reaction temperature or by the addition of the swelling agents. The changes in the pore shape and size can result in the alteration of the specific surface area, specific pore volume, pore diameter, pore length and other physical surface parameters. The changes in the pore shape and size can result in the alteration of the surface area, volume, diameter, pore length and other physical parameters.

#### 3.1.2 Hard templating methods

Pores can be introduced in inorganic materials through the hard templating process in which porous scaffolds are used as templates. Mostly, nanoporous materials with 2D and 3D porous structures prepared using SDAs are used as the hard templates. Various methods such as impregnation, adsorption and pore-filling are adopted for filling the pores in the template with the required silica or other precursors [121]. The removal of the template following the pore filling and further processing yields the final structure of the material. Silica, metals, metal oxides, carbon, polystyrenes, calcium carbonate (CaCO_3_) are generally used as the hard templates for obtaining various nanoporous structures wherein the morphology, pore size and size of the nanoparticles can be controlled by varying the morphology, the pore size and the particle size of the hard templates (Figure 1C) [116,122–124]. Multiple novel mesoporous materials including boron nitride, boron carbon nitride, carbon nitrides, metal nitrides, polymers, fullerenes [124–133] and biomolecules in addition to silica and carbon have been prepared using mesoporous carbon or silica as templates [124,134], revealing the versatility of the process of hard templating.

#### 3.1.3 Chemical precipitation

Another popular method for the synthesis of porous nanomaterials is the chemical precipitation method. In this method, a precipitate is obtained by the interaction of the dissolved precursors through a chemical reaction induced by chemical interaction between the reactants, usually aided by a precipitating agent. The particle size, pore size and structure can be controlled by the addition of surfactants in the solution. Using this approach, porous silicas were synthesised, wherein sodium silicate, ammonium chloride and CTAB were used as a silicon precursor, a precipitating agent and the porogen, respectively [135]. The porous hydroxyapatites with rod-shaped morphology and the particle size of ~10 × 50 nm in width and length were synthesised by this method, depicting the versatility of the method in achieving porosity with anisotropic shape [136]. Similarly, porous nano Nb_2_O_5_ modified with sponge-like and multi-folded nanostructures was also prepared using the chemical precipitation method [137]. These examples show the versatility of the chemical precipitation method in the synthesis of porous nanostructures with different chemical compositions, which could be used for drug delivery applications.

#### 3.1.4 Microemulsion method

The emulsion or reverse micro emulsion is another common method used in the preparation of nanoporous materials [138,139]. This is a wet synthesis process in which water-in-oil or oil-in-water immiscible but continuous aqueous-oil phases are used for the confinement of reactants during the synthesis process. The highly orderly arranged micelles in this bilayer biphasic liquid system can significantly control the formation of monodispersed nanoporous materials. For example, porous quaternary chitosan nanoparticles containing paclitaxel nanocrystals were prepared by emulsification technique followed by a crosslinking method [117,140]. Similarly, the microemulsion method was used for the synthesis of hollow and yolk/shell core silica nanospheres. The use of an oil-in-water emulsion using Triton as a surfactant, hexanol as a co-surfactant and TEOS as a silica precursor resulted in the synthesis of monodispersed particles (Figure 1D) [117]. The microemulsion method not only helps in the synthesis of monodispersed particles but also in creating smaller sized particles as well as avoiding agglomeration. For example, porous ceria (CeO_2_) was synthesised by microemulsion method using an aqueous-heptane system and cerium chloride or cerium nitrate as a Ce precursor. The surfactants were used for creating the porosity and avoiding the agglomeration of the porous ceria particles [141]. In another study, porous silica nanoparticles with a size range between 6 and 11 nm were prepared by using oil in water emulsion at different solution pH [142]. These studies demonstrated that microemulsion is a simple but effective approach for the preparation of porous nanoparticles with the controlled size and shape, which are useful for drug delivery applications.

#### 3.1.5 High-temperature methods

High-temperature synthesis methods, including the auto-combustion method, are an alternative approach for synthesizing nanoporous materials, especially porous metal oxides. A mixture of metal precursors as oxidizers and others as fuels for combustion is heated on a hot plate until it dries and then ignites by auto-combustion resulting in the synthesis of nanoporous materials [143]. The particle size can be controlled by varying the fuels present in the process and the other parameters like temperature and stoichiometry of precursors [144]. The commonly used fuels in the synthesis of metal oxide powders are urea and glycine. Through this approach, hydroxyapatite nanotubes were prepared from porous anodic aluminium oxide using Ca(NO_3_)_2_ · 4H_2_O and PO(CH_3_O)_3_ as precursors and ethylene glycol as the fuel. The mixture displayed an auto combustion behaviour at low temperature and was composed of hexagonally arranged hydroxyapatite with uniform size, diameter and length [145]. In another report, porous magnesium oxide with rod-shaped and granular morphology was synthesised by the combustion method from magnesium nitrate and ethylene glycol. Similarly, various other porous metal oxides were prepared by this route using different oxidisers and fuels [146].

Even though there are many methods available for the synthesis of porous inorganic materials including silica, metallosilicates and metallophosphates [147–157], soft templating-based approaches are more popular for the synthesis of ordered nanoporous/mesoporous inorganic nanomaterials. All these methods have their unique advantages and disadvantages. For example, the soft and hard templating methods can offer highly ordered porous nanoparticles with very high surface area and further provide the ability to tune the textural properties of the nanomaterials with a simple adjustment of the nature of the soft or hard template. However, the template removal from the porous materials is a tedious process, especially for the hard templating processes, and the scale-up is also quite challenging for these processes. Similarly, chemical precipitation and auto-combustion methods are scalable and straightforward, but the monodispersity and the textural parameters cannot be controlled through this process. Tight control over the size, shape and surface characteristics of nanomaterials is important as it has direct implications on the delivery and stability of the nanomaterials for drug delivery. Especially for lung cancer therapy, the required stability of the nanomaterials can be different for inhalation and systemic modes of administration.

### 3.2 Role of physicochemical properties of nanoparticles in the drug delivery in lungs

**Scheme 5.**
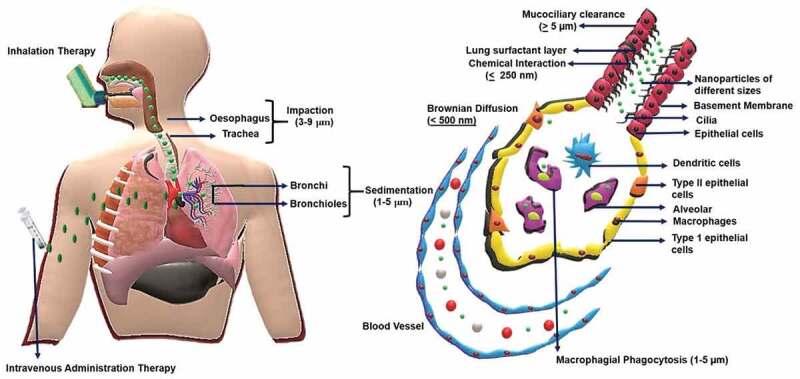
Size dependent distribution of particles in lungs. Schematic representation of size dependent deposition of particles in lungs during the inhalation therapy (adapted from [158] (Copyright 2014) RSC Advances, Royal Society of Chemistry).

It is important to ascertain the properties of nanoparticles synthesized by the above mentioned processes as the efficient delivery of the drugs using nanoporous delivery carriers depend on various factors, including the surface and textural properties of the delivery medium through a detailed description is out of the scope of this review. A thorough understanding of the physicochemical properties such as size, shape and surface charge is critical in designing nanoparticles or nanoporous particles for delivering drugs for lung cancer therapy is highly essential (Scheme 5). In the lungs, other than the common nanoparticles internalisation pathways of pinocytosis and phagocytosis, they can also be internalised through the plasma membrane diffusion method [159]. As the engineered nanoparticles have different sizes and shapes, their absorption can occur through different mechanisms based on a combination of the physico-chemical characteristics of the particles and the types of administration routes in the lungs. Hence, the size, shape and pore size of the nanoparticles are considered as the critical parameters in the design and development of drug delivery materials.

The design of nanoporous materials for lung cancer therapy is dependent on the route of drug administration. For larger doses, the relative bioavailability and the sustained plasma concentration of the drug are essential, which can be directed through the route of administration [160]. The orally administered drugs have to pass through the gastrointestinal system while the intravenous administration directly injects the drug molecules into the systemic circulation. Inhalation-based drug delivery therapies are mainly aimed at the burst release of drugs into the tumour-specific site instead of a slow, sustained release [161,162]. Reports show that the inhalation route of drug administration can reduce the systemic side effects because of the direct and fast absorption in the epithelium of the lungs [162]. In another perspective, the burst release of these drugs through the inhalation route could compromise the effectiveness of the treatment by causing lung toxicity to some extent [163]. On the contrary, slow diffusion of the drug is mainly aimed at the treatment of tumours in mucociliary pathways, especially in the upper airways, which demands more retention time [164]. In the following sections we will discuss the use of various nanoporous materials with different size, shape and morphology for drug delivery in lung cancer using different routes of administration that are affected by the factors described above.

## 4. Nanoporous drug delivery carriers in lung cancer therapy

### 4.1 Inorganic nanoporous materials

Among various classes of nanoporous materials, inorganic nanoporous materials are a unique class of materials that promise highly versatile modes of drug delivery with the potential of combining diagnostic and therapeutic features. Their stability, unique physical and chemical properties, tunable surface functionalities and versatile synthetic strategies, and morphological properties can help address the drug delivery issues alongside of lipid-based and polymer-based nanoparticles with minimal short-term and long-term side effects. In addition, the introduction of porosity and tuning the pore size is much easier in inorganic nanomaterials as compared to the organic nanoparticles. Several inorganic porous nanomaterials have been explored for lung cancer drug delivery and are reviewed in the sections below and summarised in Table 2. Among various types of inorganic nanoparticles, mesoporous silica nanoparticles (MSN) have dominated the field of drug delivery applications, including in lung cancers. Thus, this section gives more details about the MSN, its surface functionalisation, and its application in various types of therapies for lung cancer. Other inorganic nanoporous materials are discussed based on their relevance and the available literature.

**Table 2.** Major in vitro preclinical evaluations of various porous nanomaterials, advantages, limitations and future prospective are given.

Category	Drug/other molecules	Formulation components	Size, shape and porosity	In vitro results	Limitations/ prospects	Non-porous comparison models	Ref
Inorganic nanoporous materials	Doxorubicin (DOX)	Porous silicon + superparamagnetic iron oxide + CaCO_3._	Irregular morphology. Pore size – 42 ± 6 nm	Acquired 77% tumour reduction with minimal toxicity	The effect of calcium deposition has not been studied.	Nil	[231]
		MSN with ADH-1 with HA linkers	100 nm size Pore size- 2 nm	Enhanced cellular uptake	In vivo analysis has not been done		
	Cisplatin	MSN loaded cisplatin		IC50-13.8 µm	Cisplatin toxicity doesn’t reduce. Nitric oxide modification or other functionalization is another option	An inhalation model has been proposed with the same system	[233]
	PTX	Core-shell silica nanoparticles	200 nm size with a pore size of 5.79 nm	High dissolution rate for the	Reported high dissolution rate. Without proper gate blockers, lead to fast release of the drugs into the system		[234]
	Cetuximab+DOX+gefitinib	Mercapto functionalised MSN	Pore size- 2.5 nm	Low expressed EGFR cell targeting	The drug release rate and amount of drug intake is not explained	Nil	[235]
	Carfilzomib + docetaxel	MSN immobilised system	160 nm	Biphasic release system	The controlled release profile is not conclusive with the data		[194]
	Bortezomib + MMP9 inhibitor	MSN immobilised system	140 nm	Two inhibitor peptides are used	The cytotoxicity based on which peptide was not explained		[197]
	5-Fluorouracil	Folate modified Zinc based MOFs	100 nm	Drug loading is 21.3% which is 4 times higher than other reported polymer nanoparticles. Stability- 96 hrs	Further in vivo investigation needs to confirm the bioavailability of the MOFs	Nil	[236]
		Ca-Al-LDH		IC50 Values are 2.5–4.3-fold lower than normal drug	Lesser drug loading compared to other porous particles	Nil	[214]
Inorganic nanoporous materials	siRNA	Porous silicon + pegylation	Irregular morphology. Average pore size – 26 nm	Loading efficiency- 16.3 µg siRNA	The effect of calcium deposition has not been studied.	Nil	[223]
Organic nanoporous materials	DOX	GE11 surface activated liposomes with DOX	Not given	Faster Endocytosis	No cytotoxic study for GE11 has been provided with liposomes alone	Nil	[237]
		HA and CD44 modified mesoporous graphitic carbon	230 nm	50% drug loading capacity. Highly stable	Low yield		[238]
	Duanomycin	Apoferritin with CD44	28 nm	High targeting ability A natural molecule with less side effects	Easy clearance due to the small size. Hybrid conjugates has to be formed will give better results		[239]
	Methotrexate	Chitosan polymer with AS1411 targeting aptamers		Faster dissolution with targeting	Less drug adsorption	Inhalation with different chitosan in lung cancer has been proposed	[240]
	Camptothecin	Dextran porous nanoparticles		5 days of controlled drug release profile. Drug loading Percentage-11.8%		Drug percentage in high respirable fraction of 37%	[241]
	Indomethacin	Polyglycerol adipate nanospheres	160–220 nm	Drug Loading %- 25	Only In-vitro studies have been conducted. No pharmacokinetic study is provided		[242]
Hybrid Nanoconjugates	Indocyanine green+ Erlotinib	Zinc QDs modified MSN	236 nm	Dual stimuli responsiveness	Nil		[243]
	Ruthenium + H1299.2 Targeting peptide	Liposome conjugated MSN	Ru loading %- 14. Peptide loading %- 26.68 µg/ml	Higher tumour efficiency than individual liposomes and MSN	The toxicity of protein immobilised liposomes is not evaluated for comparison		[244]
	Docetaxel	Chitosan modified nanorods	90 % delivery in 48 hours				[245]
	DOX	Functionalised porous selenium	50% cell viability	Pharmacokinetics and Toxicity analysis should be conducted			[246]

#### 4.1.1 Mesoporous silica nanoparticles

MSN is a class of nanoparticles that offer unique properties like high surface area, tunable pore sizes and pore volume, high drug loading capacity and high encapsulation efficiency superior to or similar to other porous nanoparticles [165]. MSN can be classified according to the size, shape, and origin. These include SBA-1 (Santa Barbara Amorphous-1), SBA-15, SBA-16, MCM-41 (Mobil Composition of Matter No. 41), FDU-12 (Fudan University-type mesoporous material-12), KIT-6 (Korea Advanced Institute of Technology-6) and KIT-5 and other types. These different types of silica differ in the size and geometry of the pores that allow the loading of materials of different sizes. In addition to the above classification, MSNs are also categorised based on the morphology and structure such as hollow MSN, solid MSNs and different core-shell structures. MSN is used to carry various drug candidates and can be further functionalised with different moieties on its surface to achieve targeting or imaging functions. In terms of toxicity, recent studies have demonstrated that MSNs rapidly decompose in liver and are excreted renally [166,167]. It is also interesting to note that the MSN can be aerosolised using multi-exposure apparatus within the respirable size range of 0.1–3.0 µm [168]. Depending upon the synthesis and post-synthesis treatment methods, MSN can have different amounts of surface silanol groups present on its surface. While the presence of surface silanol is advantageous for the functionalisation of silica, such as attachment of targeting ligands or conjugation of drugs using stimuli responsive bonds, surface silanol groups can interact with the phospholipid layers of the blood cells and lead to haemolysis [169]. These surface silanol groups also provide versatility to the MSN by functionalisation of various imaging and targeting agents for multifunctional attributes.

##### 4.1.1.1 Functionalisation of MSN

**Scheme 6.**
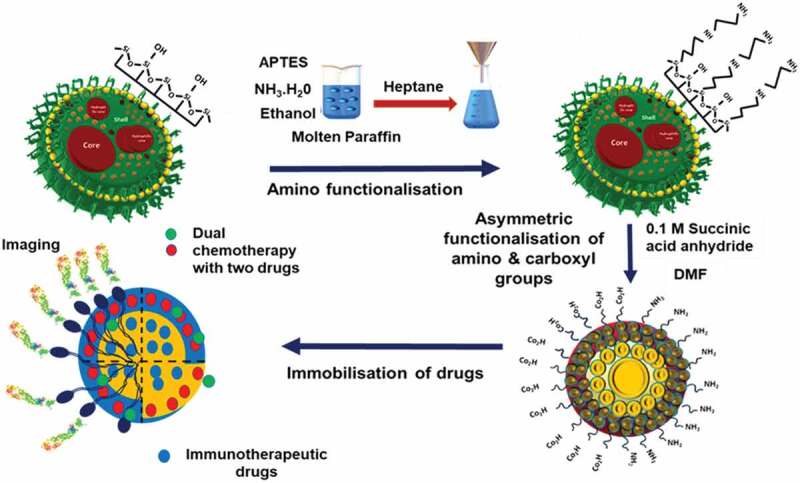
Functionalisation of silica nanoparticles. Schematic depiction of the dual drug loading and functionalisation of mesoporous silica nanoparticles.

Current drug delivery approaches demand a carrier that can deliver the drug in the targeted area with longer blood circulation and retention time. To achieve the desired properties, the current generation of drug delivery carriers is modified by functionalizing the surface to attach various agents for imaging, tracking, targeting, and releasing drug molecules. In addition, the surface functionalization presents an opportunity for stimuli-responsive drug delivery by the use of gate-keeper molecules or by attaching drugs covalently to the surface of particles using specific stimuli-responsive bonds that can be broken in the presence of external or internal stimuli to release the attached drugs. In this respect, MSNs with a high density of surface silanol groups have the edge over all other materials. The surface silanol groups can be directly functionalised with various groups or can be converted to more suitable functional groups, such as thiol, amine and carboxyl, for further attachment with ligands and/or drugs. Scheme 6 depicts a strategy that can be used for attaching two different surface functional groups. This strategy can be further used for the conjugation of two different entities, such as a drug and a fluorescent agent. In addition, the advancements in silane chemistry have resulted in the availability of various types of silanes with terminal functional groups. These silanes can be co-condensed during the synthesis of silica or can be grafted on the surface post-synthesis of silica, giving even greater flexibility for the functionalisation of MSNs.

Two types of procedures are used for the surface functionalisation of MSNs. Post-synthetic grafting is the process of surface modification of porous silica nanoparticles after their synthesis. Functionalisation with the desired functional groups on the surface of MSNs is achieved by silylation, especially with organotrialkoxysilanes like TEOS (tetraethyl orthosilicate), APTES (aminopropyl triethoxy silane), GPS (glycidoxypropyltrimethoxysilane), orthosilicic acid, silicic acid or organotrichlorosilanes [170]. The silanol groups present on the external surface of MSNs are more kinetically accessible for functionalisation than other silica nanomaterials, and the post-synthetic grafting is usually performed after the surfactant removal [171]. Co-condensation is another functionalisation strategy for grafting functional groups on the surface of MSNs. It is an adaptation of the sol-gel chemistry between tetraalkoxysilane and one or more organoalkoxysilanes such that both the functional silane and tetraalkoxylsilane condense together during the sol-gel process [172,173]. The organosilane condenses to form the core structure of the MSN and the functional silane co-condense on the surface results in the formation of terminal functional groups. At present, a majority of research on drug delivery and cellular uptake using MSN is based on functionalised MSN [174–176]. Functionalisation of MSN can change its drug adsorption and release characteristics, stability in aqueous media and also influence the uptake efficiency. Hence, it is important to study their behaviour post functionalisation. Selected recent reports using functionalised MSN are discussed below.

Functional groups like primary amine, organothiol, and methyl functional groups were grafted onto MSNs through different silane derivatives and analysed the uptake efficiency of MSNs [165]. Similarly, Tomoiaga and coworkers functionalised amino groups on the surface of MSNs to study the drug adsorption efficiency. Different drug adsorption and uptake behaviours were expected after attaching these multifunctional groups owing to the difference in the properties of functional groups. For example, mercaptopropyl is more hydrophobic as compared to APTES. It was observed that the functionalisation with thiols and amines increased the drug loading efficiency after the functionalisation. An organic functionalised SBA 15 having primary amine, organothiol and methyl group showed a slightly higher amount of antibiotic amoxicillin adsorption as compared to amine functionalised SBA 15 of 27.5% from 18.3% [165]. Functionalisation of (2-(butylaminoethyl)) glycine groups on the HMSN was achieved by co-condensation method, which showed an increase in entrapment efficiency of cisplatin from 1.6% to 31.1% without changing either the morphology or size [177]. The glycine functionalisation avoids the interaction of cis-platin with other functional groups such as carboxyls and increases the drug loading capacity.

**Figure 2.**
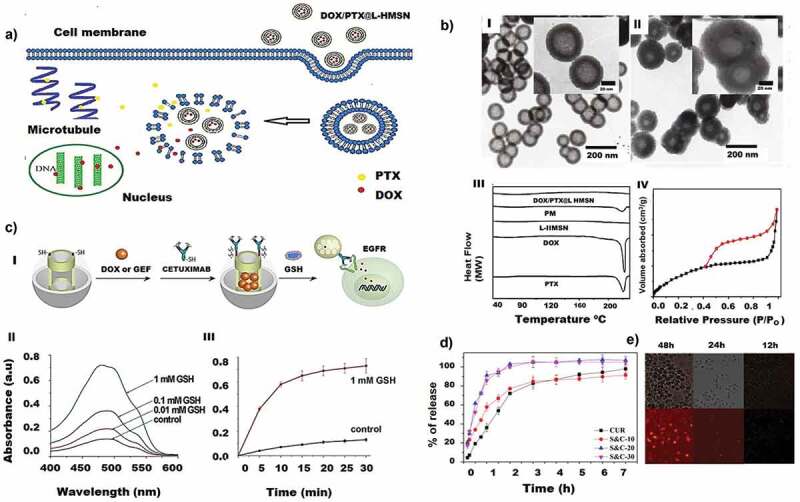
Silica nanoparticles for drug loading and release. A) Illustration of DOX/PTX loaded lipid coated HMSN and corresponding cellular uptake. B) (I) TEM images of HMSN; (II) L-HMSN; (III) The DSC profiles of PTX, DOX, L-HMSN, PM (DOX:PTX:L-HMSN = 9:1:12 (m/m/m)) and DOX/PTX@ L-HMSN; (IV) The N_2_ isotherms of HMSN. C) (I) Schematic illustration of CET modified MP-SiO_2_ loaded with DOX and gefitinib. (II) GSH stimulated spectra of the released DOX from CET-capped MP-SiO_2_ NP and the (III) Time-dependent spectra of the DOX release from CET-capped MP-SiO_2_ NP. D) The CUR release behaviour of composite particles. E) Phagocytosis of SA-15 by RAW264.7 cells at different times. (Reproduced with permission from 2A, 2B [178]. (Copyright 2017) Material science and Eng: C Elsevier, 2C [179]. (Copyright 2016) Scientific reports, Nature, 2D, 2E [183]. (Copyright 2019) European Journal of Pharmaceutical Sciences, Elsevier).

Other than attaching inorganic moieties, MSNs surface can also be modified with lipid coating which is represented in Figure 2A. The presence of a lipid layer on the surface of MSNs showed less toxicity with the high drug loading efficiency of DOX and PTX [178]. These phospholipid coated MSNs have a size of 202 ± 11 nm. The hollow portion adsorbs water-soluble DOX through physical adsorption while the PTX loaded lipid layer blocked the pores by completely covering the surface through the lipid film hydration method. Stable encapsulation efficiency of 41 ± 5% with DOX and 4.5% paclitaxel on the surface was reported. Even though a high drug encapsulation efficiency was achieved, as shown in Figure 2B, the total available drug was only 4.5% of the loaded drug, resulting in a smaller drug release.

Surface modification of MSNs is used for attaching targeting agents for the targeted delivery of drugs to drug-resistant or mutant lung cancer cells. A typical study targeting EGFR (Epidermal Growth Factor Receptor) mutant lung cancer cells with MSNs has been conducted. The surface of MSN was modified and functionalised with the targeting drug cetuximab (CET), a tyrosine kinase inhibitor specifically targeting the EGFR, and the MSN pores were loaded with DOX and gefitinib for giving a dual therapy (Figure 2C) [179]. The surface of the MSN with a specific surface area of 887.9 m^2^/g was functionalised with 3-mercaptopropyltriethoxysilane to introduce mercapto group. The MSN was loaded with DOX and gefitinib and was further conjugated with CET by cross-linking of disulfide bonds. The drug release occurred with the cleavage of sulfur bond due to the presence of abundant glutathione enzyme (GSH) within the tumour cells followed by the release of chemo drugs (Figure 2CII, 2CIII). CET capped MSNs in the EGFR resistant PC9 cells showed higher endocytosis than in low EGFR expressed BEAS-2B cell lines, ascertaining the effect of CET functionalisation in improving the endocytosis.

As discussed above, surface functionalization has become a key part of the development of silica-based nanomaterials for drug delivery applications. It allows flexibility to attach imaging and targeting agents to the surface of silica nanoparticles. However, the surface functionalization may also block the pores or change the drug loading and release characteristics if not done correctly. Some of the challenges associated with surface functionalization are the quantitative analysis of surface functionalization and the control of the functionalization process to attach multiple drugs or targeting agents. Despite this, it has become an integral part of the silica-based system that is used for drug delivery applications, including all forms of chemotherapy, radiotherapy and gene therapy, as discussed below.

##### 4.1.1.2 Chemotherapy

MSNs have been used for delivering chemotherapeutic drugs for the treatment of various types of cancer. In lung cancer treatment, MSNs have been used alone or as hybrids combined with other imaging and targeting functionalities. Cisplatin is a popular drug for the treatment of lung cancer. However, its concentration and size-dependent toxicity severely limit its efficacy. MSNs have been tried as a drug carrier for cisplatin. A recent study showed an IC50 value of 13.8 µM in A549 cell line. Despite the high drug loading in the MSN, the platinum toxicity was not reduced [180,181]. This was compensated in another study where the cisplatin cytotoxicity was reduced significantly after nitric oxide (NO) modified MSNs were loaded with cisplatin. The use of NO can sensitise the cells and reduce the cytotoxic effect of cisplatin on normal cells. For the NO conjugation, the amine functionalised MSN were subsequently converted into N-diazeniumdiolate NO donors (NO-AMS) via exposure to NO pressure (60 psi) and cisplatin was absorbed by AMS [182].

Immobilisation of natural molecules has also been tried with MSNs to study the drug immobilisation efficiency and the delivery capability of a nanoporous system. Curcumin (diferuloylmethane) is a natural polyphenol that is derived from turmeric (curcuma long L.). One of its disadvantages is poor water solubility that severely limits its bioavailability. Curcumin was loaded in SBA 15 by wet impregnation method and showed a high drug loading (~72%) as well as drug release (26.2%) (Figure 2D) from the SBA 15 within an hour. It was argued that the solubility of the curcumin might be improved due to the change of the crystalline state of curcumin to an amorphous state. However, the supporting information provided to prove a change of crystalline state to an amorphous state was quite limited. In addition to the antitumour activity in both in vivo and in vitro studies, the aerosolized SBA-15 curcumin loaded drug delivery could also reduce the metastasis with minimal side effects (Figure 2E). Thus, it can be considered as a potential tool for lung cancer therapy [183]. Previous studies have indicated that PEGylation and conjugation with lipids can enhance the uptake of nanoparticles, increase their circulation time and minimize the individual cytotoxicity [184].

##### 4.1.1.3 Phototherapy

**Figure 3.**
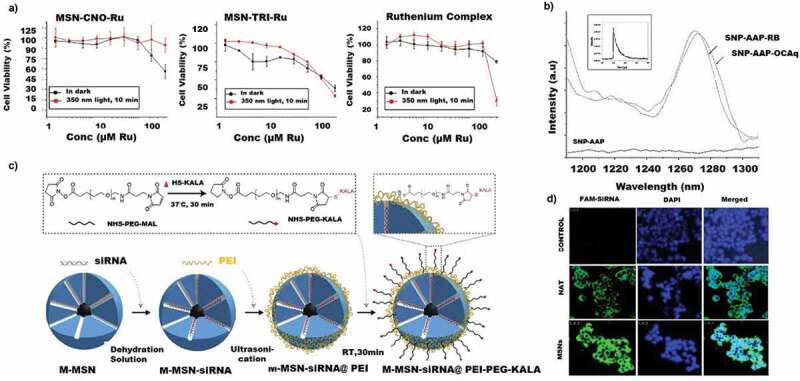
Mesoporous silica nanoparticles used for phototherapy, targeted therapy, chemo and gene therapy. A) In vitro cytotoxic analysis of MSN- RU in A549 cells (dark and 10 min light irradiated conditions. B) Phosphorescence emission spectra of singlet oxygen (^1^O_2_) measured for SiNP-AAP, SiNP-AAP-RB, SiNP-AAP-OCAq×10x (inset shows the lifetime measurement). C) Schematic representation of MSN KALA conjugated peptide. (D) Confocal microscopy studies of intracellular uptake of FAM-labelled si-RNA (NAT and MSN formulations) at 6 h in A549 cells. (Reproduced with permission from 3A [188]. (Copyright 2010) Dalton Transaction, Royal Society of Chemistry, 3B [189]. (Copyright 2016) Journal of Photochemistry and Photobiology B: Biology, Elsevier, 3C [193]. (Copyright 2014) Biomaterials, Elsevier, 3D [194]. (Copyright 2018) ACS Appl. Nano Materials, American Chemical Society).

Phototherapy (including photodynamic and photothermal, PTT/PDT) being a targeted therapy, is becoming increasingly popular due to its reduced side effects and localised mode of action. MSNs have been used as carriers to photosensitise molecules in several cancers, especially lung cancers [185,186]. By integrating within MSNs, the aggregation of photosensitiser molecules can be reduced, which in turn increases the anti-cancer efficiency. MSN functionalised with ruthenium(III) complexes were recently tried for PDT and corresponding sensitisation [187]. Upon light irradiation, the ruthenium complex sensitises and kills the tumour cells. To attach the photosensitive polypyridyl ruthenium(II) complex using triamine, a three step functionalisation strategy was adapted. At first, the 3-chloropropyltriethoxysilane was functionalised on the surface of MSN followed by the functionalisation of 3-isocyanatopropyltriethoxysilane. This was followed by a final modification of tris(2-aminoethyl) amine and formed triamine-functionalised MSN. More research to confirm the ruthenium toxicity and metal deposition in the body has to be conducted to progress these metal-based studies further. Other than metal functionalisation, phospholipid functionalised MSN has also been utilised for selective photodynamic therapy in lung cancer cells. A nanoformulation consisting of phospholipid coated MSNs loaded with photosensitiser protoporphyrin IX (PpIX) and further functionalised with folate targeting agents has been experimented in lung cancer cells. To attach the PpIX ligands, the MSN surface was transformed to hydrophobic by treating with 13-(Chlorodimethylsilylmethyl)heptacosane (CDSMH) that helped to attach the hydrophobic porphyrin [188]. The phospholipid capping resulted in creating stable nanoparticle dispersion at a pH of 7.4 in PBS. The incubated concentration of PpIX loaded in the phospholipid modified MSNs was 10 µM. The fluorometric analysis (λ_ex_ = 401 nm; λ_em_ = 605 nm) showed a six-time higher abundance of PpIX embedded in MSN in A549 cells than free PpIX. The in vitro cytotoxicity data were not as promising (Figure 3A), suggesting a lack of photosensitization achieved by this formulation.

Phototoxicity studies of common drugs loaded on MSNs have also been initiated in lung cancer cells. Anthraquinone are a group of molecules that are used as malarial drugs. 9,10-anthraquinone-2-carboxylic acid, chemically bonded to the surface of MSN functionalised with 3-(2-aminoethylamino) propyl (AAP) group, showed phototoxicity in A549 cells under visible light irradiation. The high phototoxicity observed in this study was ascribed to the generation of singlet oxygen (Figure 3B) that promoted apoptosis [189]. The deep tissue treatment using phototherapy suffers from the lack of light penetration. Hence, the probes of for phototherapy have shifted from the UV light towards the NIR emitting probes, which enables the photothermal therapy [190]. Similarly, other advance Fluorescence Resonance Energy Transfer (FRET) based investigations are deemed to be safer and stable comparative to other thermal response [191,192]. As noted, the aforementioned studies are in the semi-advanced stages of research that needs to be extended to in vivo/preclinical studies to understand their translation as the release rate of the drugs and the anti-cancer efficiency would be different in the living system.

##### 4.1.1.4 Targeted chemo- and gene therapy

Biomolecules such as proteins, siRNA, mRNA or DNA are large sized molecules that are hard to encapsulate in non-porous or microporous drug delivery carriers. Usually, these molecules are embedded or attached on the surface with surface agents of non-porous carriers. These attached molecules can be easily lysed by the enzymes within the biological environment. MSNs with large pore diameters are ideal for encapsulating and delivering of such large biomolecules that are impermeable to cell membranes, especially for cancer therapy.

MSN loaded with siRNA for targeting vascular endothelial factors (VEGF) was studied by Chen et al [193]. The siRNa was loaded in the core by the co-dispersion method. This siRNA loaded MSN was capped by polyethylenimine (PEI) for further functionalisation and polyethylene glycol (PEG) acts as an anti-fouling coating. Finally, the MSN was conjugated with a fusogenic KALA peptide with endosomolytic function (Figure 3C). The nanocarriers showed negligible cytotoxicity at 200 µg/ml in A549 cells and LO2 cells; however, the siRNA loaded MSN showed severe toxicity in A549 cells and low toxicity at 50 µg/ml in LO2 cell lines The cytotoxicity in A549 was higher than the commercially available lipofectamine™. This confirmed the strong action of the siRNA loaded MSN, which was due to the higher transfection efficiency in the metastasised lung cancer cells owing to the high loading and targeting efficiency of the functionalised MSNs.

A similar attempt was made to deliver the drug carfilzomib (a proteasome inhibitor) along with the two other drugs such as etoposide and docetaxel, immobilised in MSN for lung cancer therapy. This three-cargo loaded MSN was also functionalised with a siRNA with a monodisperse size of 160 nm (Figure 3D). A biphasic release of the drugs from functionalised MSN was observed due to the hydrophobic nature of both the immobilised drugs resulting in high cytotoxicity in lung adenocarcinoma cells [194]. Similarly, a hollow MSN loaded with DOX, was functionalised with ADH-1 (vascular targeting cyclic pentapeptide) using hyaluronic acid (HA) as a linker for dual targeting into the lung cancer cells. ADH-1 can block the function of N-cadherin, while HA can target the CD44 receptors on the tumour cells. This dual-targeting resulted in inhibiting the tumour cell invasion by down-regulating the expression of N-cadherin and depicted higher cytotoxicity compared to the non-targeted therapy [195]. In another approach, the MSN were used as a synergistic inorganic nanohybrid tool. The MSNs were converted to ‘janus’ nanoparticles by two different surface modifications. MSNs were functionalised with HA and DMMA (2,3-dimethylmaleic anhydride) on either side by a pickering emulsion method and keeping the size of nanoparticles less than 100 nm. The modified janus MSNs were able to target the CD44 receptors in the lung cancer cells by the HA ligand and the DMMA molecules initiated a charge reversal at the acidic pH of A549 cells resulting in a higher uptake by the cancer cells [196].

Bortezomib (BTZ) is a clinically approved proteasome inhibitor for different cancer treatments. The main drawback of this medication is the reduced solubility similar to cisplatin. BTZ loaded MSNs modified and hybridized with histone H2A peptide showed better drug delivery in lung cancer cells. Histone H2A is a chimeric peptide that can overcome targeting obstacles in drug delivery. On comparing these two studies, the MSN modified with targeting agent is more specific with better efficacy [197]. Similarly, van Rijt et al. reported the MSNs capped with avidin (a tetrameric biotin binding protein) and functionalised with matrix metalloproteinase inhibitor 9 (MMP9) linkers. The linkers are peptide sequences which can be cleaved by overexpressed MMP9 in the cancer cells. The drugs, BTZ and cisplatin were loaded separately, and the efficiency of MSN as a delivery agent was compared. It was found that in lung cancer cell line A549 and H1299, both the drugs were released only in MMP9 expressed cell lines [198].

Other than MSNs, more silica structures can be noted in the literature. Silica nanorattles have been proposed as a drug delivery carrier by Sundarraj et al. for targeting cytosolic phospholipase A_2_α cPLA_2_α [199]. cPLA_2_α is an enzyme-linked for cell cycle regulation and controls arachidonic and eicosanoid expression of inflammation and cancer. The irregular level of these enzymes is a marker of several lung cancers, including NSCLC. Silica nanorattles were developed to encapsulate an enzyme pyrrolidine-2, an inhibitor of cPLA_2_α. This inhibition reduces systemic toxicity and further enhances therapeutic efficiency for lung cancers. Pyrrolidine could specifically target the cytosolic phospholipase A2 (cPLA2α) along with EGFR antibody and offer better targeting specifically to the tumour site, resulting in a higher uptake of the nanorattles than non-targeted nanorattles [199]. In comparison to MSNs, this study did not characterize the nanorattles completely before and after the pyrrolidine-2 loading and the morphological changes observed due to such functionalization. However, the nano rattles depicted a distinct core and a shell with a total size of 86 nm diameter that can be used for further studies in future research. Moreover, the synthesis of nanorattles is simple compared to that of other core-shell MSNs with mesoporous shells.

As described in the examples above, MSNs are at the forefront of drug delivery technologies for the treatment of lung cancer. The highly tunable pore sizes and pore geometry have also been used for loading two different drugs for combinatorial therapy in order to achieve better efficacy. Even though MSNs have been used in many studies with systemic administration, inhalation therapies with MSNs and their targeting modalities are not yet fully explored and are a potential area of future research.

#### 4.1.2 Other porous nanoparticles

##### 4.1.2.1 Core-shell nanoparticles

Core-shell nanoparticles (CSNs) are a category of promising drug delivery carriers for lung cancer therapy. Core-shell structure offers a bimodal pore distribution, consisting of an inner core and an outer shell for drug accommodation without compromising the surface area or the pore structure and depict a low mass transfer resistance in the blood vasculature [200]. The core-shell structures can be of different variations, including multi-core, multicomponent core, multiple shells, porous shell and even smaller or larger core attached externally or internally to a single shell. CSNs are versatile NPs that can be chemically modified to different forms like hydrophobic core and a hydrophilic shell or vice versa by utilizing different polymers and synthesis strategies.

Among the CSNs, the most experimented particles in lungs are the silica-based core-shell nanoparticles, mostly in the form of hybrids, conjugated with other nanoparticles [201,202]. The CSN can be designed with a single core [203] or multiple cores or with multiple shells [204] or hybrid conjugates like metal nanoparticles with silica [205], metal oxide nanoparticles with silica or mesoporous carbon cores with or without metal-doped modifications [206–208]. The particle size, shape, pore size, pore volume and surface properties can be tuned and modified according to the need of cargo loading and types. Due to the versatility of core-shell structure, it has been utilized to achieve multiple drug delivery functions such as chemotherapy, radiotherapy, gene therapy and imaging or tracking functions.

A nano formulation with an improved dissolution rate of drug paclitaxel has been reported by immobilisation within core-shell silica nanoparticles [208]. The core-shell structure was synthesised with a size of 200 nm and a specific surface area of 585 m^2^/g, pore volume of 0.33 ml/g, and average pore size of 5.79 nm. These good surface properties allowed a high paclitaxel loading of up to 46 ± 1% (PAC-csMSN). A high dissolution of 86 ± 3% for CSN encapsulated paclitaxel was observed, which is comparatively higher than normal paclitaxel (28 ± 4%) within 1 hour of drug administration to the A549 cells. The high dissolution rate is correlated with the high dispersion of the drug on the mesoporous shell. Even though the toxicity results were significant, the drug release profile of the nanoparticle was not satisfactory, depicting a burst release in the initial 1 hour that shows a lack of the controlled release. CSN has also been used to administer radiotherapy in lung cancer. There are similar reports available on a non-porous core-shell structure developed with a core made of gadolinium oxide and a shell of polysiloxane with functional moieties of diethylenetriaminepentaacetic acid (DTPA). However, the efficiency of the radiotherapy in the in vitro studies was not satisfactory and required further investigation for more clarity [209,210].

**Figure 4.**
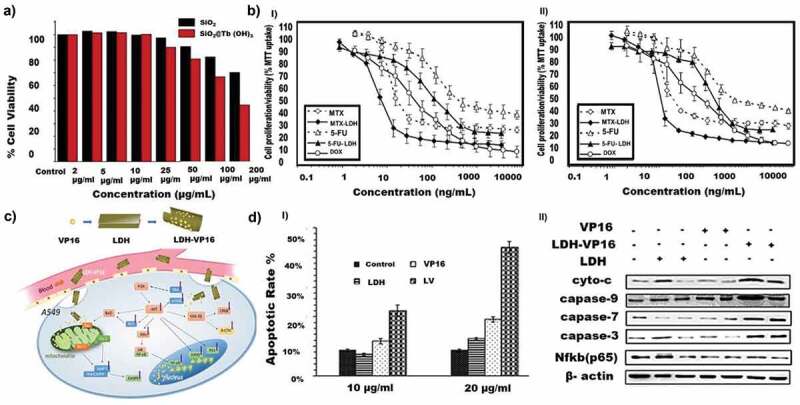
Cell viability and uptake of core-shell nanoporous materials. A) Concentration dependent cytotoxicity by MTT assay in A549 cells treated with SiO_2_ and SiO_2_@Tb(OH)_3_ at 2–200 mg/ml for 24 h. Results are presented as mean ± SD from three independent experiments. B) A comparative drug sensitivity of 5-fluorouracil incorporated LDH in Hep1 (I) and A549 (II) C) Schematic illustration of LDH-VP16 targeting mitochondria and inhibiting P13K-AKT signalling pathways on NSCLC. D) Results on VP16 and LDH-VP16 induced apoptosis on A549 cells (I) Percent apoptotic ratio of A549 cells (II) Western blots depicting the protein content. (Reproduced with permission from 4A [212] (Copyright 2019) Colloids and Surfaces A: Physicochemical and Engineering Aspects, Elsevier, 4B [214] (Copyright 2008) Journal of Physics and Chemistry of Solids, Elsevier, 4C, 4D [219] (Copyright 2015) Acta Biomaterialia, Elsevier).

Nano-scale luminescent lanthanide materials are gaining much interest in cellular imaging due to their high quantum efficiency, long decay, and superior photochemical stability. The drawback of this material is that it has weaker emission, which suppresses its luminescence efficiency. To overcome these limitations, Hao et al. constructed a hybrid chiral core−shell nanostructure including a UCNP (NaYF_4_:Yb^3+/^Er^3+^) core and a chiral NiSx NPs-decorated MOF shell (denoted ‘UCNP@ZIF-NiSx’). The material demonstrated ultrasensitive and selective detection of ROS species (H_2_O_2_) in live cells and in vivo [211]. Similarly, Ansari et al. synthesised mesoporous core-shell silica nanoparticles and functionalised them with terbium III hydroxide via the co-condensation method in the basic media of NaOH [212]. The presence of terbium III hydroxide was confirmed with the XRD, and FTIR. A concentration-dependent cytotoxicity was observed for the terbium loaded CSN. This study can be utilised by combining imaging with other satisfactory combinations of drugs to tackle drug delivery (Figure 4A).

CSN has also been used for the delivery of genetic materials. Researchers have modified CSN with the positively charged proteins for attracting DNAs. An advantage of such a strategy is that it can enhance the total DNA condensation and nuclear localisation in cancer cells. To achieve a targeted therapy, Rong et al. designed a gene delivery platform synergising BTZ with histone H2A-hybrid cationic peptide along with upconversion guided mesoporous CSN. The drug was loaded in the mesopores and the gene p53 peptides/H2A was functionalised on the surface of MSNs. In this case, the core contains upconverter photoluminescent particles coated with a shell of CTAB, and the H2A were functionalised by EDC/NHS-mediated grafting reaction [198]. These materials could achieve a higher 4.17 fold increase of the relative transcriptional level of p53, which is confirmed with the qRT-PCR assay. Thus, a synergetic effect of transfection of p53 gene to the p53 null NCI-H1299 cells with the drug BTZ could be achieved and initiated apoptosis of the mitochondria-mediated pathways. This is an initial study to initiate strategies for delivering the genes to any cells/tumours.

Although there are several advantages noted for the MSN, including CSN, only selected studies are available in the literature, and a comprehensive assessment of these nanoparticles is still lacking. Larger in vivo studies in small animals and primates will help to assess the efficiency of the system for drug loading, tracking and release within the system.

##### 4.1.2.2 Layered double hydroxides (LDH)

Layered double hydroxides LDH, are a class of materials characterized by a layered structure similar to clay minerals. It is usually formed from the mixture of divalent and trivalent metal hydroxides which are orderly arranged in alternate layers with interlayers and the space filled with anions and water molecules [213]. Compared to other inorganic materials, LDH has been less explored as a drug delivery vehicle in lung cancer therapy. One of its limitations is the dependence on electrostatic charges for drug loading. Thus, most of the drugs are loaded in the LDH by ion-exchange methods. Choi et al. loaded 5-fluorouracil in the magnesium aluminium LDH [214] and tested its effect on various cancer cell lines including the lung cancer cell lines. The successful incorporation of 5-fluorouracil was ascertained from the interlayer spacing that corresponded well with the size of 5-fluorouracil. The drug incorporated in LDH showed higher sensitivity to A549 and liver carcinoma cells (Hep1) cells as compared to the free drug (Figure 4B).

A facile synthesis of CaAl-LDH (calcium aluminium LDH) nanoparticles and a simple anion exchange technique to load drug etoposide into Ca-Al-LDH showed a synergistic effect of both tumour reduction and suppression of CAMKIIα expression with SOD gene activity in the lung cancer cells (Figure 4C). It is known that free etoposide administration has several side effects such as acute toxicity to healthy cells, peripheral neuropathy and strong inflammatory response at the injection site. At the molecular level, CAMKIIα expression and SOD gene activity indicate the inflammation and toxicity levels of the drug response. After 24 hours and 72 hours of incubation of etoposide-LDH nanoparticles in A549 cells, there was a significant growth inhibition of 21.56% (confirmed by apoptosis) in cancer cells along with a reduction of CAMKIIα expression (95.14 pg/ml) and a four-fold reduction in SOD gene activity (Figure 4 DI, II) [215].

The disadvantage of the LDH based drug delivery vehicles in lung cancer is that the incorporation of large drug molecules and neutral drugs within the layers is very difficult. This is due to the restricted surface area and the limited interplanar spacing between the inner layers. It is also observed that the pulmonary surfactant can easily destroy the layered structure of LDH and thus, the drug delivery efficiency is very poor in the case of lung cancer models. Even though several hybrids have been reported and tested in other cancers [216,217], only a few studies are reported in LDH nanoparticles [217,218]. Addressing these drawbacks by constructing a hybrid platform will be required for the utilization of LDH as drug delivery vehicles for lung cancer or any cancer therapy.

##### 4.1.2.3 Porous silicon

**Figure 5.**
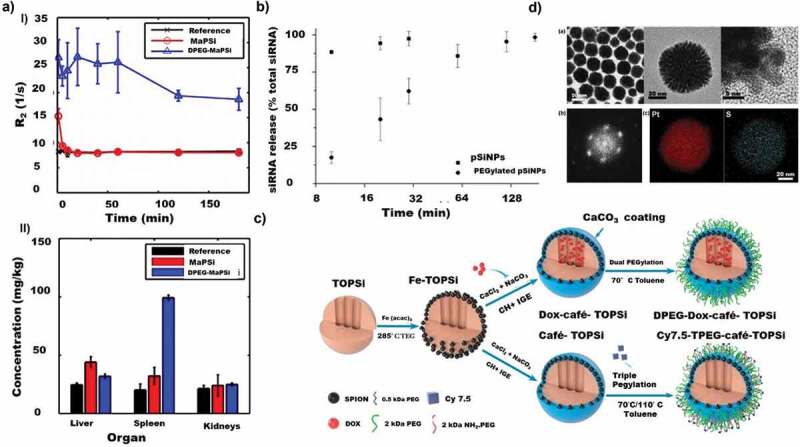
Porous silicon for lung cancer treatment. A) I) Measured T2 relaxation rates (R_2_, mean ± std, n = 3) of 5% mannitol injected samples with nanoparticles for the plasma samples taken from the animals after the nanoparticle injection. The injected samples were reference (black crosses), the MaPSi (red circles), and the DPEG-MaPSi (blue triangles). II) ICP-MS analysis of the silicon content of the organs (mean ± std, n = 3), in the reticuloendothelial system. The samples were taken and analysed after 3 hrs of injection. B) siRNA release profile of pSiNPs and P-pSiNPs, respectively. C) Schematic illustration of particle modification routes. Oxidized PSi (TOPSi), DOX loaded Fe-TOPSi nanoparticles with calcium carbonate coating (DOX-CaFe-TOPSi). Dual PEGylated PEG (DPEG-DOX-CaFe-TOPSi). D) TEM images of porous PtNPs. (Reproduced with permission form 5A [222]. (Copyright 2016) ACS Appl. Mater. Interfaces, American Chemical Society 5B [223]. (Copyright 2014) Journal of Nanoparticle Research, Springer, 5C [231]. (Copyright 2019) International Journal of Pharmaceutics, Elsevier, 5D [232]. (Copyright 2019) Biomaterials, Elsevier.

Porous silicon (pSi), as the name suggests, is a form of elemental silicon-containing a porous structure. pSi is usually synthesised by an electrochemical perforation etching method or metal assisted chemical etching method [220]. pSis formed by electrochemical etching depicts high porosity with a surface area between 200 and 300 m^2^/g, with a size less than 500 nm [221]. It has several promising characteristics for drug delivery. pSi nanoparticles are biocompatible with minimal side effects, as they decompose into orthosilicic acid over a period in the body. However, this biodegradable and biocompatible material has disadvantages such as lower colloidal stability, less retention time in blood circulation, and unstable behaviour in both in vitro and in vivo systems. In 2016, Nissinen et al. reported a functionalised pSi with dual PEGylation (DPEG), which increased the circulation half-life of the nanoparticle from 1 to 241 minutes in the lung mouse models (Figure 5A) [222]. A 10 nm thick DPEG coating was achieved by utilizing silane coupling chemistry. Similarly, Nakki et al. moved a step further by modifying the pSi with both magnetic and pH-responsive agents and loaded chemo drug DOX in the pSi platform followed by triple PEGylation. The pore-blocking ability and pH responsiveness of CaCO_3_ was combined with the iron oxide magnetic nanoparticles.

pSi is mostly given via the systemic administration and the detailed investigations of pSi as an injectable nano vector either in the form of suspension or powder are rare. The potential application of pSi for molecular targeting has also been explored in various ways through systemic administration. The specific synthetic small interfering RNA (siRNA) which targets (M2 isoform of pyruvate kinase, PKM2) the glycolytic pathway of lung cancer cells was loaded on the surface of the pSi. Loading the siRNA into the pSi reduces the drawbacks associated with direct siRNA delivery, like sensitivity to nuclease degradation and reduced permeation in cells due to its negative charge. Loading of siRNA on the surface of the pSi was achieved after the PEGylation of the surface by electrostatic adsorption. 95% of the loaded siRNA was released within 30 minutes of the administration, indicating a burst release profile (Figure 5B) that could be reduced by PEGylation [223]. Promising results on albumin coated pSi for the drug delivery of paclitaxel also substantiate the use of pSi as a drug delivery carrier in lung cancers [224]. It was claimed that the albumin coating increased the diffusion resistance and decreased the dissolution rate of pSi.

Despite the current work on pSi-based drug delivery in lung cancer, there are drawbacks in the system. pSi depicts a fast dissolution rate in the physiological conditions limiting its potential for slow release of drugs for a sustained period. Even though silicon is an essential trace element for the bone and collective tissues, the surface groups like Si–H, SiH_2_, and SiH_3_ can easily form reactive silanes [225–227] that could invoke an immune response. The high solubility of the porous silicon can further cause dose-dependent toxicity [228]. Other issues such as the lack of *in vivo* studies detailing the drug delivery efficiency, uptake efficiency, degradation, and toxicity profile also limit the advancement of the pSi as the drug delivery agent to clinical trials.

##### 4.1.2.4 Metal-organic frameworks and other metal hybrids

Biomedical applications of metal-organic framework (MOFs) are rapidly expanding due to their unique properties such as tunable pore size, high surface area and the possibility of attachment of different functional groups. They are fabricated by self-assembly of metal ions and polydentate bridging ligands through different synthesis routes like, solvothermal method, rapid precipitation method, one-pot synthesis, reverse microemulsion, a rapid microwave-assisted method and ultrasonic synthesis [229]. When compared to other porous nanoparticles, MOFs have advantages such as multifunctional properties with small crystal density and controllable pore size. There are certain inherent issues with MOFs, such as low structure stability in physiological media with a low degree of drug internalization. A proper choice of cation and an organic linker modification can make them suitable as a drug carrier [230]. Appropriate modifications on the MOFs with various materials will significantly reduce these drawbacks.

Several hybrids of silica, MSNs, pSi, metal oxides and polymers in different morphologies, including core-shell assembly, have been explored for lung cancer treatment [231–246]. In these applications, the role of the base matrix is to load the drugs while a second component is added to give versatility to the system by either adding a secondary drug, a controlled release agent, or a stimuli responsiveness to the system. A hybrid system consisting of a pSi core with a silica shell surrounded by iron oxide nanoparticles covered with CaCO_3_ layer and finally shielded with dual PEGylation PEG (DPEG-DOX-CaFe-TOPSi (Figure 5C) showed an excellent therapeutic effect in lung cancer adenocarcinoma cells and in vivo studies [231]. The CaCO_3_ coating created an inter pore cavity of size 80 nm that enabled a high (10 ± 1 wt%) drug loading of the model drug DOX in the hybrid structure. Conjugation of pSi with CaCO_3_, reduced the unnecessary release of the drug in the body, which in turn reduced the cytotoxicity of the drug. Further, the addition of iron oxide nanoparticles enabled MRI imaging and tracking of the hybrid nanoparticles. While the reduction in tumour size was similar for free DOX and the DOX encapsulated within the hybrid, the significantly higher systemic toxicity of free DOX was reduced by protecting DOX in hybrid nanoparticles is a promising development.

Porous metallic nanoparticles have also been used for drug delivery. For example, porous platinum nanoparticles have been developed for effective and enhanced radiotherapy treatment. The porosity was introduced in the metal system with the help of a soft templating method using cationic surfactant CTAB and the reduction of platinum using ascorbic acid followed by the PEGylation with -SH bond. The resulting structure showed a well-defined porous structure with a particle size of 65 ± 7 nm (Figure 5D) and uniform distribution of platinum. The in vitro toxicity of the material in the NCI-H460 (human NSCLC cell line) and the in vitro therapeutic efficacy of x-ray irradiation in presence of Pt nanoparticles was further analysed. It was reported that the high-Z radio sensitizing ability of Pt maximizes the radiation dose delivered to the lung cancer cells. A treatment with an X-ray dose of 0, 1, 5 and 10 Gγ showed an enhanced anti-cancer effect of porous Pt in cancer cells as compared to the normal Pt mediated irradiation effect [232].

The success of porous inorganic materials has been demonstrated in both in vitro as well as in vivo applications. Some of the prominent studies with in vivo testing illustrating the advantages of inorganic nanocarriers and other materials for treating lung cancers are summarised in Table 3. Understanding the advantages and drawbacks of these inorganic nanocarriers in vivo can give an outlook on future research. Core-shell designed PTX loaded-MSNs (PAC-csMSN) were tested for their in vivo pharmacokinetics, and it was found that there was an improved plasma retention. However, the researchers did not include the therapeutic efficacy of such formulation, making it difficult to understand their future applicability for lung cancer [234]. A study on the efficiency of hollow MSN in lung cancer has been conducted by loading with BTZ. In vivo study on H1299 xenograft showed improved efficacy and better results with P53 gene therapy. However, this study could have included a combination therapy using P53 gene therapy to confirm their final assumption [197].

**Table 3.** Major in vivo preclinical evaluations of various porous nanomaterials, advantages, limitations, and future prospects are given.

Category	Drug	Formulation components	In vivo highlights	Limitations/future prospects	Ref
**Inorganic nanoporous materials**	PTX	Core-shell structured PTX-mSiO_2_ NPs (PAC-csMSN)	Pulmonary absorption increased for PTX and could be beneficial for the lung cancer treatment	No therapeutic study has been done using the formulation. Therefore in order to make it for further application, it is necessary to understand their in vivo fate	[234]
	Bortezomib	Hollow mSiO_2_ nanospheres for bortezomib encapsulation to improve efficacy for lung cancer	Improved efficacy against H1299 tumour xenograft	Better anti-tumour efficacy would have been obtained if HMSNs-BTZ were administrated in combination with gene therapy using wild-type P53	[197]
	5-FU	Inhalable Mesoporous Silica NPs for curcumin delivery to lung cancer	The mesoporous inhalable silica /Curcumin NPs showed improved effects on B16F10 melanoma metastatic lung mouse model	Long term toxicity studies could have been benefitted for the public understanding of this formulation	[247]
	5-FU	5-FU loaded Ca(II)-MOF	Inhibited lung cancer cell progression, invasion and migration in vivo	No safety and PK analysis given	[248]
Organic nanoporous materials	PTX	PTX Nanocrystals combined with porous Quaternized Chitosan NPs	Orally administered NPs showed improved effects on lung cancer cells	Why oral administration is preferred over intravenous injection was poorly explained	[249]
	voriconazole	Pulmonary deliverable PLGA) nanoparticles containing voriconazole for	Porous NPs with lower (mass median aerodynamic diameter) MMADs had improved pulmonary deposition and sustained presence in lungs.	In vivo therapeutic efficacy was not studied	[250]
	DTX	DTX-loaded chitosan microspheres	Targeted drug delivery system for Lung cancer	Lack of therapeutic analysis in vivo	[207]
	miR-660 (Micro RNA NPs)	Lipid-NPs entrapping miR-660	Improved therapeutic effects on PDXs (patient derived Xenografts) with no side effects in normal cells and tissues.	No specific in vivo limitations	[251]
Hybrid nanoconjugates	CPT	rGO/Carbon/mSiO_2_ Nanocookies for CPT release	NIR‐responsive local and on-demand multi-modal therapeutic system for combined chemotherapy/hyperthermia	No studies for in vivo toxicity of the hybrid nanomaterials. Since most of the hybrid nanosystems are not FDA approved it is important to make sure that they are non-dangerous to the human body	[252]
	DOX	Hybrid multifunctional nanoparticles consisting of porous silicon, superparamagnetic iron oxide, CaCO_3_, DOX and polyethylene glycol	The study highlights the effective strategy of hybrid porous nanoparticles on cancer cells	*Biodistribution study was conducted on lung cancer cells, whereas the therapeutic efficacy conducted using CT26 cells*	[253]
	DOX	Porous silica hybridized with PNIPAAm for DOX delivery to lung carcinoma cells	DOX delivered within lung carcinoma cells by Radiofrequency assisted magnetic hyperthermia with improved effects in 2 weeks	Long term toxicity studies are not given	[254]
	Afatinib	Powdered porous PLGA microspheres for codelivery of Afatinib-Loaded Solid Lipid NPs and PTX	PK/PD analysis on Sprague–Dawley rats showed Afatinib and PTX can retain 96 h of high lung concentration without causing harm to other major organs, ensuring their safety	Therapeutic efficacy analysis could have been done for more understanding of this formulation	[255]
	Genistein and All-Trans Retinoic Acid	Lipid/Protein Core-shell hybrid NPs for Genistein and all-trans retinoic acid delivery	Inhalable spray-dried Nano powders shown improved lung deposition and higher therapeutic benefits on lung cancer model	Lack of long term toxicity	[256]
Functionalised nanoporous materials	Gefitinib	Cetuximab-modified mSiO2	Improved therapeutic effects on PC9-DR xenograft tumours via endocytosis of large amount of nano-medicine and the effective gefitinib release induced by high GSH level in PC9-DR cells.	Detailed long term toxicity is not available	[235]
	EGFR	EGFR conjugated mSiO2	Improved lung cancer targeting	Significant Improvements both In Vitro and In Vivo	[257]
	Myricetin	FA-modified mSiO_2_ NPs in combination with MRP-1 siRNA	Improved suppression effects of myricetin on lung cancer by specific and selective intratumoural accumulation	Long term toxicity could have been studied	[258]
	DOX	TPGS‐modified Polydopamine‐ mSiO_2_ for enhanced lung cancer chemotherapy against multidrug Resistance	Improved lung cancer therapeutic benefits	No explanation has been given for the improved lung accumulation from TPGS‐Functionalised Polydopamine‐modified mSiO_2_	[259]

In another attempt, inhalable curcumin loaded composite particles based on MSN showed better results on metastatic lung cancer models. On converting the MSN nanocomposite into the aerosolised capsule, the percentage of fine particle fraction (FPF) has increased to 57.3% and a progressive release of curcumin to the lungs from 0.25 to 1 hr maximum up to 26.2% was achieved. The B16F10 melanoma metastatic lung mouse model was used for this study and these NPs had improved therapeutic benefits after insufflation once every day. The in vivo study showed an improved antitumour reduction with a lesser inflammatory response. However, the insufflation method could have been compared with other administration techniques (example: Intravenous) for a better conclusion [247]. The administration of 5-FU loaded Ca(II)-MOF synthesised using an ultrasound-assisted synthesis inhibited the human lung cancer progression by inducing cell apoptosis. The main drawback in this study is the lack of safety and pharmacokinetic (PK) analysis for these newly developed MOFs [248].

Based on the discussion, it is found that porous inorganic nanomaterials have opened a tremendous opportunity for the delivery of drugs owing to their multifunctional attributes, ease of grafting and versatility to combine drugs with proteins, imaging agents, drugs and other therapies such as radiotherapy, phototherapy and hyperthermia. The challenges associated with inorganic nanomaterials are centred on their distribution and clearance from the body. The biocompatibility of many of the inorganic carriers except silica and iron oxide is also a subject of discussion and porous organic materials, especially polymers and lipids, have a slight advantage over inorganic carriers in this regard, as will be discussed in the next section.

### 4.2 Organic Nanoporous Materials

Organic materials are more often used in drug delivery applications than inorganic nanoparticles due to their high biocompatibility, low cost and less cytotoxicity with minimal immunogenicity. The major currently researched porous organic particles and their recent progress is reviewed in this section.

#### 4.2.1 Liposomes

Liposomes were one of the first nanoparticles approved by the FDA as drug delivery vehicles in cancers. Liposomes or lipid nanoparticles are organic nanoparticles formed with vesicles encapsulated by concentric lipid bilayers. Liposomes comprise of molecules such as phosphatidylcholine or cholesterol. The aqueous core and the lipid membrane has the capability to transport both hydrophobic and hydrophilic drugs. Since their inception, these particles have evolved with various modifications and have been a part of several clinical trials, including lung cancer treatments. Many anticancer drugs have been incorporated within liposomes, although these usually incur the problem of a burst release due to intermediate solubility of the drug and high partition between liposome bilayers. Similarly, the liposomes platform is considered superior for water-soluble drugs as it can encapsulate these drugs in its aqueous cavity; however, the loading of hydrophobic drugs is limited due to the limited space between the hydrophobic tails of the lipid bilayers [260]. The liposomes have been used for lung cancer research through targeted therapy. As an organic nanoparticle, any hybrid conjugates associated with the liposomes are very relevant to discuss in relation to porous materials.

**Figure 6.**
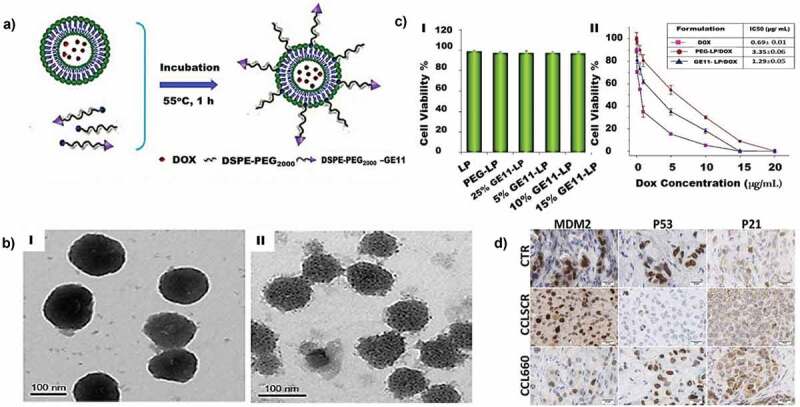
Different organic nanoparticles for antitumor studies. A) Schematic illustration of DOX loaded liposomes containing GE11 (B) and TEM images of DOX loaded liposomes (I) and DOX-loaded liposomes containing GE11 (II). C) (I) Cytotoxicity of the plain liposomes, PEG-LP, and GE11-modified liposomes with different GE11 densities. (II) Cytotoxicity of DOX, PEG-LP/DOX, or GE11-LP/DOX in A549 cells at different DOX amounts D) MDM2, P53 and P21 IHC expression on PDX treated with CCL660 or CCLSCR or CTR (n = 4 for CTR, n = 5 for CCLSCR and n = 6 for CCL660). Figure 6a, 6b, 6c : reproduced from reference 213 (adapted from [262] (under CC BY 4.0). International Journal of Nanomedicine, Dove Press, 6D [279]. (Copyright 2019) Journal of Controlled Release Elsevier.

Second generation DOX loaded liposomes (Doxil) has been approved by the US Food and Drug Administration as a medication for various cancers [261]. Cheng et al. reported a targeted chemotherapy approach using liposomes by loading DOX into liposomes and activating the surface of liposomes with a novel GE11 peptide for targeting EGFR receptors in A549 cell lines which is described in the schematic representation of Figure 6A and the internal structure was confirmed with TEM (Figure 6B) [262,263]. A 90% conjugation efficiency of GE11 and liposomes was confirmed by HPLC. The targeted and drug-loaded liposomes showed a 2.2 fold higher drug release and tumour growth inhibition in A549 cells than free DOX. To confirm the effect of GEII peptide modified liposomes, EGFR negative K562 tumour cells were also incubated with the GEII-LP/DOX (Figure 6I, II), which showed no response to the drug delivery system. The drawback of this study was that the cell viability of the GEII-LP/DOX is very poor, even in normal cells. Although the cytotoxicity is reduced after PEGylation, further research is required to confirm the GEII ligand targeting in lung cancer cells.

Tumour site penetration with the drug delivery carrier system is the most problematic task, especially in relation to organic nanoparticles. Administration of liposomes was explored by immobilizing the drug triptolide (diterpenoid epoxide) into the liposomes functionalised with dual ligands. The dual ligands functionalised on the surface were anti-carbonic anhydrase IX (anti-CA IX) and CPP33 (tumour lineage-homing cell-penetrating peptide, with a sequence of RLWMRWYSPRTRAYG). MTT assay showed that the triptolide loaded liposomes modified with dual ligands has a higher toxicity than the bare liposomes without modification. The results were confirmed in both the 2D cell lines and in 3D tumour spheroids [264]. Octreotide molecule is another targeting agent that mimics the natural somatostatin and enables the liposomes to easily bind with the surface receptors on cancer cells as well as escape the macrophage degradation. The octreotide functionalised liposomes were loaded with the drug epirubicin with an encapsulation efficiency of 97.8%. The survival rate of lung metastatised cells treated with peptide modified liposomes was reduced significantly than normal epirubicin encapsulated liposomes [265]. Cao et al. designed macrophage membrane coated and emtansine loaded liposomes with high expression of α4β1 integrin for specifically targeting the metastatised lung tumours from the breast cancer cells [266]. These macrophage membrane coated liposomes were able to bind to the overexpressed 4T1 sites on the metastasised tumour cells and specifically target the same.

Multifunctional targeting with minimal toxicity can be achieved by various surface modifications; however, majority of the studies in this area are conducted as in vitro studies in lung adenocarcinoma. Subsequently, only limited studies have explored the pulmonary delivery of liposomes for lung cancer therapy [267]. There are minimal studies about toxicity or sedimentation and systemic accumulation of liposomes via inhalation. This is an area of significant research potential, and further studies are required to explore the liposomal role in lung cancer.

#### 4.2.2 Synthetic Polymers

Polymers have been well explored in drug delivery research for drug conjugation and release in relation to lung cancer research. The stability and toxicity of polymeric nanoparticles in lung cancer delivery are still at the validation stage. Certain macro and micro polymers have shown high deposition rates in lung cancer tissue due to their suitable aerodynamic size [268]. In this section, we review some of the porous polymers that have been explored as drug delivery carriers for the treatment of lung cancer.

Polylactic-co-glycolic acid (PLGA) is a co-polymer composed of lactic acid and glycolic acid which is utilised as a drug delivery carrier for systemic administration. This highly biocompatible and biodegradable polymer was approved by the FDA and the European Medicines Agency [269]. Unlike other polymers, the surface charge of the PLGA is negative, which requires further surface modification with cationic polymers for attaching genetic materials. Compared to non-porous polymer nanoparticles, porous PLGA can load both hydrophilic and hydrophobic drugs, which is an added advantage.

PLGA immobilised with DOX and PTX has shown promising results in lung cancers with high intake efficiency [270,271]. Kim et al. designed highly porous micron-sized PLGA particles functionalised with a peptide TRAIL (tumour necrosis factor (TNF)-related apoptosis-inducing ligand) and labelled with fluorescein N-hydroxysuccinimide for the delivery of DOX. TRAIL is reported to have no toxicity because it specifically binds to the overexpressed receptors such as DR4/TRAIL-R1 and DR5/TRAIL-R2 in cancer cells, which are not present in normal cells. The mean loading efficiency of DOX and TRAIL in PLGA microparticles was 87 ± 7% and 92 ± 2%, respectively. The synergistic effects of TRAIL modified DOX loaded PLGA were analysed in both in vitro (H226-human lung squamous cells) and in vivo mouse models administered via the inhalation route. The DOX was gradually released and sustained in the cells up to 7 days. However, the TRAIL leached out within a day, and only a trace amount was retained by the seventh day. The toxicity of DOX and TRIAL were analysed in H226 lung squamous cell carcinoma and it was found that co-treating with DOX (~1 µg/ml) and TRIAL (~0.01 µg/ml) reduced the maximum inhibitory concentration (IC50) of DOX of 3 µg/ml with 0.01 µg/ml TRIAL concentration [68].

In a similar study, Feng et al. modified PLGA particles by immobilizing two drugs, DOX and PTX in 5:1 ratio suggesting that this dual chemotherapy drug combination with targeting ligands will be a promising area of research in lung cancer in future [272]. Another targeted approach involving micro RNA therapy using PLGA microparticles by encapsulating DOX, modified with polyethyleneamine/miR-34a (which is a microRNA regulated by P53) was reported. Transwell migration assay and wound healing assay were used to confirm the potential ability of PLGA loaded with DOX and miR-34a to inhibit cell proliferation. As confirmed by flow cytometry, targeting and drug-loaded PLGA microparticles showed a higher inhibitory effect by inhibiting the G2 phase and activating caspase-mediated apoptotic pathways (37.76% and 37.26%). The inhibition of cell migration was higher in the PLGA microparticles treated cells as compared to the direct administration of DOX [273].

Extensive in vivo studies have also been carried out for PLGA nanoparticles [274–277]. For example, PLGA NPs (207–605 nm) containing voriconazole (VNPs) was tested for its pulmonary deposition in swiss albino mice using a customized inhalation chamber. The detected drug content in the lung was higher (7 days) compared to the control non-porous NPs (5 days). The major limitation of this study is the lack of therapeutic effects using the developed NPs. Since porous NPs with the lower mean aerodynamic diameter (MMADs) showed better pulmonary deposition and sustained presence in the lungs, they could have had a better therapeutic benefit. Therefore, the future direction of this study should be focused on therapeutic benefits and long-term toxicity profile to make sure that these NPs are safe to go for a clinical application [250].

PLGA porous microsphere dry powders for codelivery of afatinib-loaded solid lipid nanoparticles and PTX had improved therapeutic benefits for EGFR Tyrosine Kinase Inhibitors Resistant NSCLC. PK/PD (pharmacokinetics/dynamics) studies using Sprague–Dawley rats showed a 96 h lung retention of afatinib and PTX without associated organ comorbidities, confirming in vivo safety. The initial findings confirmed that these NPs might be beneficial, especially for cancers with MDR characteristics. The main drawbacks of this study are the lack of long-term safety analysis and in vivo anti-tumour studies [232]. Similarly, docetaxel chitosan microspheres showed improved pharmacokinetics in Kunming Strain mouse (5 mg/kg formulation with DTX), but no therapeutic analysis was carried out [278]. Cationic lipid nanoparticles entrapped micro RNA NPs (CCL660) showed improved efficacy on PDX Lung tumours with no immune toxicity. Figure 6D represents the long-term treatment effects on the PDX Lung tumour model [279].

Similarly, other polymeric nanoparticles have been reported for the delivery of chemotherapeutics drugs, for example, porous organic polymers for delivering the drug quercetin. The use of organic polymers has intrinsic difficulties in relation to metabolism and pre target biological accumulation, and these were addressed by attaching an acetal bond that completely solubilises the particles in acidic media [280]. Other than polyesters, polyanhydrides are also studied in localised cancer therapies, especially as wafer type of insertion, which can provide a continuous stream of drug release [281]. These porous polymeric nanoparticles showed less cytotoxicity with high drug loading capacity; thus, the introduction of porosity will allow for a vast range of drug delivery.

#### 4.2.3 Natural Polymers

Bionanoparticles with porosity are derived from naturally inspired molecules that bind with organic or inorganic nanoparticles for several structural and chemical functionalities. These self-assembled natural protein complexes are obtained from biological sources, which have the potential to reduce the side effects caused by anti-cancer drugs [282,283]. Unlike in other cancers, a very few studies have been reported in the treatment of lung cancer using porous bio nanoparticles, which are discussed in this section.

Apoferritin is a self-assembling 24 polypeptide subunit that stores iron and assembles into a protein nanocage with an internal diameter of 8 nm and an external diameter of 12 nm. Removal of iron core from this protein cage resulted in the formation of a hollow nanoparticle suitable for drug delivery applications. Apoferritin molecules dissociate at a pH of 2 and self-assemble at neutral pH conditions. Thus, the drug can be released in a pH-dependent manner without any modifications with less toxicity [284]. Luo et al. immobilised the anticancer drug daunomycin (DN) (a drug from anthracycline family, mainly used in acute myeloid leukemia) into apoferritin nanocages (DN-Apo) by a simple diffusion method at a pH of 5. The daunomycin is a small molecule that depicts side effects such as nephrotoxicity and neurotoxicity. The DN-Apo was further modified by attaching the hyaluronic acid (HA) which can target CD44 receptors and further modified with PLAA (poly-l-aspartic acid) through peptide–protein interaction. APO-DN-HA-PLAA depicted higher tumour inhibition than pure DN. The total size of the hybrid after modification of peptide and PLAA is 28 nm which will be a challenge for the deposition of protein cage in lung cancer especially via pulmonary administration [239].

Natural polymers are also explored as a drug delivery carrier in lung cancers. Most of the natural polymers are composed of polysaccharides or proteins. The disadvantage of these naturally made polymers is that the strength of the molecules might rapidly degrade physiological conditions, which can cause premature release of the loaded drug. The most researched natural polymer in lung cancer is chitosan, a cationic amino polysaccharide, derived from partial deacetylation of chitin. Chitosan-based microspheres have been used for the administration of various vaccines and even as drug delivery vehicles via inhalation routes [240,285]. From a pharmacological perspective, the chitosan microspheres are better than apoferritin, in which the stability of the bionanoparticles can be lost during aerosolization. Different forms of chitosan particles have been used in lung cancer for drug delivery. A partially quartenernised derivative of chitosan, N-((2-hydroxy-3-trimethylammonium) propyl) chitosan chloride (HTCC) has been used in lung cancer drug delivery by Lv et al [249]. Enhanced chemotherapy is reported by using chitosan nanoparticle conjugated with nucleolin-targeting aptamer AS1411 and immobilised with the drug methotrexate for lung cancer therapy [286]. In both studies, encapsulating both drugs with chitosan enhanced the transportation across the intestinal barrier to the target site.

Using short interference RNAs (siRNA), targeted therapy for various pathways has been studied with different nanoparticles such as polymers, inorganic and organic nanoparticles. Challenges, including poor conjugation, reduced stability, and ineffective release to the cytoplasm, must be overcome before a successful translation of siRNA-based nanomedicines. To overcome these challenges, Suresh et al. developed gelatin nanoparticles functionalised with antibodies [287]. Gelatin is another natural molecule made up of collagen and is used for reducing systemic immune response during drug delivery. Gelatin was synthesised with two-step desolvation process with glutaraldehyde as a crosslinker. To reduce the toxicity of the gelatin arising from the aldehyde groups on gelatin, excess glutaraldehyde was quenched with tris glycine. The modified gelatin was functionalised with cetuximab (anti-AXL antibody) by EDC/sulfo-NHS chemistry. AXL (Ark or UFO) is a member of TAM family of receptor tyrosine kinase, which is highly expressed in cancers. The gene silencing efficacy was analysed by western blotting in EGFR mutant H820 cells. The H820 cells were incubated with the gelatin-antibody for 72 hours, and it was estimated that >25 nM of transfected-siRNA was required for ~70% knock-down of AXL protein. Thus, high transfection efficiency with minimal toxicity can be achieved by utilizing porous gelatin hydrogels. Similar studies are reported in other cancers such as breast and colon cancers [288].

Similarly, several clinical trials are progressing on the cyclodextrin polymer conjugated camptothecin as a drug delivery vehicle for SCLC. Introducing porosity in these systems could increase their drug loading capacity, and this could contribute significantly to a new area of research [289]. Meenach and Kim et al. developed camptothecin loaded acetylated dextran (AC-DEX) porous microparticles for pulmonary delivery. AC-DEX are polyesters and produce acidic by-products causing local pH change, which is a key for onsite drug release. The loading efficiency of camptothecin in AC-DEX was 11.8% and 55% of the drug was released within days of the administration, suggesting a sustained release capacity of the AC-DEX particles as compared to normal PLGA microparticles. It was also observed that the efficiency of delivering a respirable fraction of the drug is higher in AC-Dex than PLGA, thus it is a promising drug delivery polymer [241]. There are various possibilities of using natural polymers and bio nanoparticles in drug delivery, especially via the pulmonary inhalation route to the lungs. The issues of stability of the particles can be overcome by surface modification and hybridisation with different moieties. Thus, bio nanoparticles hold a great promise for future development as a drug delivery system [283].

#### 4.2.4 Nano Carbons

Carbon-based nanostructures have been explored as drug delivery carriers for various diseases. Porous carbon is of biomedical interest due to its unique attributes, namely, geometry, adjustable pore size, high surface area, abundant framework compositions, biocompatibility, and uniform interpenetrating pores. The porous carbon is categorised into solid carbon particles, hollow porous carbon nanomaterials and core shell porous carbon nanomaterials with a difference of micro, meso and macro size. One of the striking advantages of mesoporous carbon over other mesoporous materials is its pore size ranging from 1.5 nm to 10 nm [290]. By utilizing the pore size of the carbon nanomaterials, researchers have attempted to build intelligent nanocarriers for lung cancer therapies [291].

**Figure 7.**
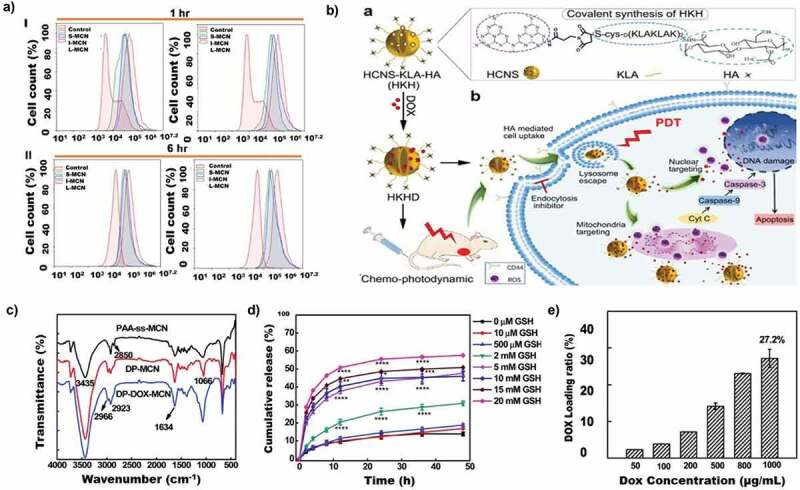
Effect of nanocarbons in lung cancer cell**s**. A) MCNs triggered a burst of ROS generation. (I) FACS analysis in J774A.1 cells in different concentrations of MCN. (II) Relative ROS levels in L-MCN-treated cells with or without N-acetyl-cysteine (NAC). B) Schematic illustration of different mechanisms of Mitochondria- and Nucleus-Targeting carbon nitrides containing KLA, HA, and DOXa. C) FI-IR spectra of PAA-ss-MCN, DP-MCN, and DP-DOX-MCN. D) Controlled DOX release profile from DP-DOX-MCN at pH 5.5 at different GSH concentration. E) DOX loading ratio on PAA-ss-MCN in 10 mM PBS buffer in a 24 hrs incunation time at a pH 8.5. (Reproduced with permission from 7A [292] (Copyright 2017) Journal of Environmental Sciences, Elsevier, 7B [293] (Copyright 2019) Molecular Pharmaceutics, American Chemical Society, 7C,7D, 7E [295] (Copyright 2017) Journal of Material Chemistry B, Royal Society of Chemistry).

A toxicity study of mesoporous graphitic carbon in lungs was initiated by Chen et al. who observed a high toxicity of the mesoporous graphitic carbon in the lung surfactant lining at a concentration of 10 µg/ml [292]. This is further confirmed with the assay to analyse the intracellular ROS production in the macrophages. As shown in Figure 7A, the ROS level increased in the cells after the treatment of 5 and 10 μg/mL concentration of MCNs at 1 and 6 hrs. However, the toxicity can be reduced by modifying the size, shape and surface coating or modifying the surface with various surface passivating agents. As a further step, Xie et al. functionalised the surface of the hollow carbon (HC) with the two targeting agents, hyaluronic acid (HA) and CD44 receptors and loaded the same with the DOX [293]. These targeting agents, shown in Figure 7B, target the mitochondria and the nuclei and enter via CD44 receptor mediated endocytosis. The surface of the HC was modified by the addition of sulfur groups by maleimide reaction followed by acetamide reaction for attaching HA. A high drug loading was achieved in the HC due to the strong π–π stacking of the graphitic plane of HC. An increase of DOX fluorescence was observed in the presence of hyaluronic acid, which confirmed the targeting potential of HA modified HC followed by the release of the DOX. The release percentage was 48% at a pH of 5.5, confirming an acidic pH dependent DOX release for treating lung cancers [293].

Masuda et al. reported-on mesoporous carbon modified by aptamer conjugation for targeting intracellular proteins in lung cancers especially glycosylated mucin 1 (uMUC1). MUCI is usually secreted in the secretory epithelial cells, but it is usually overexpressed in lung cancer, especially adenocarcinoma cancer types. Targeting these receptors using aptamers are considered as an efficient way of targeting in lung cancer cells [294]. A biocompatible mesoporous carbon was coated with polyacrylic acid/polyethyleneimine (PAA/PEI) double polymer shells and further modified with MUC1 aptamer (Figure 7C). The double polymer shell reduced the drug leakage from the mesoporous carbon. The maximum drug loading observed was 27.2% and the release rate was controlled by different stimuli, including the GSH dependent controlled release (Figure 7 D, E) [295].

The drawbacks of carbon nanoparticles are their low yield in synthesis and issues related to the purification of carbon materials [296]. Porous carbons have high hydrophobicity that limits their use for systemic administration and loading of hydrophilic drugs. Once the synthesis methodology is standardised to address the limitations of the porous carbon materials, this will be a promising drug delivery vehicle in lung cancer treatment.

### 4.3 Hybrid Nanoconjugates

Hybrid nanoconjugates are comprised of both inorganic and organic nanocarriers with various functionalities attached to their surfaces. The important rationale behind the designing of a hybrid nanoconjugate is to compensate for the drawbacks of individual nanoparticles in terms of structural and functional properties or to combine diagnostic and tracking functionality in a single drug carrier [283,297,298]. Several hybrids materials like hybrid super paramagnetic iron oxide NPs (SPIONs) and multifunctional gold NPs have been studied and evaluated in lung cancer diagnostics and extended to create theranostic functions. Some of the latest progress in the development of hybrid nanoconjugates will be critically reviewed in this section

#### 4.3.1 MSN based Hybrids for Delivery and Theranostic Applications

Mesoporous organic-inorganic hybrid materials are synthesised by coupling organic or inorganic nanoparticles via template-assisted synthesis processes. This can be achieved by subsequent attachment of inorganic or organic groups by grafting, co-condensation, one-pot synthesis or by the use of silylation and further conjugation of the inorganic/organic moieties [298].

Inorganic metal/metal oxide hybrids with MSN have been studied recently due to their stability, small molecular size and easy synthesis methods. Self-assembled zinc oxide nanocapsules have been utilised for delivering different drug molecules for lung cancer therapies. It is proven that the zinc modified, or the zinc immobilised molecules succeed in addressing several drawbacks of the drug delivery carriers or drug molecules such as drug side effects, agglomeration and long-term deposition [299]. Zhang et al. reported the efficiency of MSN-ZnO quantum dot hybrids synthesised by electrostatic interaction. Indocyanine green (ICG) was loaded into the amine functionalised mesopores with a high loading of 0.02 mg/mg of MSN. The mesopores were sealed and occupied by QDs, which were subsequently attached with the targeting drug erlotinib (ER) (targets overexpressed EGFR receptors) through coupling reactions. For a successful reaction with ER and to improve the solubility, the surface of the hybrid was intermediately functionalised with N-(4-bromopthaloyl)-chitosan before ER bonding [243]. The pH-dependent and GSH dependent dual-responsive drug delivery potential were analysed by treating the sample in different pH conditions in the presence and absence of GSH. At the neutral pH, the ZnO QDs are strongly bonded and stable enough to prevent drug leakage while at pH 5 the disulfide bonds are cleaved by the GSH, resulting in a fast release of ICG. Zn^2+^ decomposes at the acidic intracellular environment of cancer cells releasing the drug IG. The maximum in vitro cytotoxicity of the hybrid composite was observed at 25 μg/mL. Other than MSN, the anticancer activity of ZnO has been investigated in the lung cancer cells using hybrid nanocarrier synthesised with berberine (BER) & ZnO [300]. The results showed that these hybrids are non toxic and reduce the tumor proliferation in both in vitro and in vivo studies. The advantages of this hybrid system are that the individual toxicity of the ZnO and the berberine is significantly reduced by combining with laser irradiation. An individual study on the effect of nitrogen doped ZnO in small cell lung carcinoma also has been reported [301]. The nitrogen doped ZnO effectively reduced the GSH–GSSG ratio without increasing the Zinquin ethyl ester in normal cells. Zinquin ethyl ester is an indication of Zn^+^ ion toxicity. This research provides a wider opportunity to explore potential applications of ZnO nanoparticles in lung cancer treatment.

Similar to zinc, gold nanoparticles also have been designed to target several intracellular proteins for catalysing the drug release such as Se-enzyme thioredoxin reductase with high specificity [302]. A dendritic folic acid modified MSN composite has been used to deliver an anti-tumour agent, a mononuclear cyclometallated Au(III) complex with 2-benzylpyridine ligand ([Au(bzpy)Cl_2_]). The dendritic spherical MSN showed a wrinkle shaped morphology with a size of 82 ±6 nm and was able to load the gold complex at a concentration of 120 mg/L. When compared to the zinc molecules, the drug loading achieved in this study is lower, which could be attributed to the size of the molecule. As there was no pore blocking agents to control the pH mediated release, 50% of the drug was released at neutral pH. The in vitro tumour inhibition was maximised at the concentration of 200 μg/mL with lesser toxicity to normal cells than other compounds.

Gold nanorods capped chlorin e6 (Ce6) doped mesoporous silica nanorods have also been used for photosensitive and hyperthermia treatment in lung cancer cell lines. In this study, Ce6 was used to control the morphology of the MSN. The advantage of this conjugation is that it can perform dual therapy, photothermal therapy (PTT) from the gold nanorods as well as PDT due to the formation of singlet oxygen from Ce6 after the uncapping of the gold nanorods, following PTT, from the mesoporous silica nanorods surface [303]. In a different modification, conjugation of polydopamine with hollow gold core MSNs with perfluorohexane have also been studied in lung cancer cells. The surface was modified with arginine-glycine-aspartic acid (RGD) peptide via a PEG spacer for targeting and anticancer drug DOX and was loaded for combination chemotherapy. The total size of the hybrid was 182 nm with a polydopamine shell of about 15 nm that was used for multimodal imaging as well as PTT. A high photothermal ablation efficiency of 23.3% was achieved within 5 min of laser irradiation in 4T1 cells [304], suggesting a good photo response from the hybrid.

Designing a multifunctional drug delivery carrier for theranostic application is highly challenging with various obstacles. Attaching organic dyes or QDs for imaging applications using simple post-synthesis methodologies with firm binding stability has an associated high degree of difficulty [305]. Carbon dots (CD), a fluorescent carbon-based nanomaterials, have several advantages like excellent water dispersibility, high photostability and excellent biocompatibility [306,307]. CD can be used in place of QDs for imaging and diagnostic purposes. A MSNs-CDs nanohybrid with high surface area and pore volume after conjugation was synthesised and loaded with DOX as well as functionalised with folate targeted receptor. The total structural functionalities including the presence of carbon dots were confirmed by the weight loss of 12.6% from TGA and the C, N, O content from XPS. An enhanced drug release efficiency was observed at an acidic pH of 5.5 which is attributed to the reduced π-π interaction between the DOX and the MSN-CDs [308]. Even though the results confirmed the enhanced release of the drug and thus the anti-tumour efficacy, the stability of the conjugate after the drug release is not explained and thus a detailed analysis could be helpful in the future.

Organic/organometallic-based MSN hybrids have also been used for combining multiple functionalities in a single drug delivery system. The redox abilities of ruthenium-based drugs have been reported in the treatment of lung cancer with MSN based hybrids. Ruthenium III complex reduces to an active ruthenium II complex in the reductive tumour environment and targets the nuclear DNA with fluorescence. Shao et al. combined inorganic and organic nanoparticles together to achieve higher tumour suppression by combining the MSNs with protein incorporated liposomes for H1299.2 receptors of the lung cancer cells [244]. A targeting peptide, which consists of green fluorescence protein (eGFP), large-conductance mechanosensitive channel protein (MscL) was combined along with the H1299.2 targeting peptide. The ruthenium was loaded into the MSN (RU-MSN) with a loading percentage of 14.2 (measured by inductive coupled plasma optical emission). The liposome was prepared with the traditional approach and then dried, and the targeted protein was immobilised in the presence of PBS at a neutral pH of 7.4. The liposomes and the RU-MSN were combined (RU-MSN-LIPO) by electrostatic fusion with 1 hour of pipetting. The total anti-tumour effect of targeted RU-MSN-LIPO was 4.18 times higher in H1299 cells than the normal cells. Even though a higher anti-tumour effect was shown in the cell lines, the individual protein immobilised liposomes or the effect of peptide in the cell lines by experimental assays like western blotting was not discussed.

#### 4.3.2 Other porous nanoconjugates

Limited studies including other nanohybrids have been reported for lung cancer treatment. Other than the mesoporous silica nanoparticles, mesoporous nanorods have also been synthesised as a drug delivery carrier to treat different cancers especially nasopharyngeal carcinoma. 100 nm long mesoporous silica nanorods were synthesised using a slight modification in the concentration of the condensation catalyst [309]. The nanorods were functionalised with carboxyl group that was used to covalently bind to the DOX. Compared to MSN spheres, the MSN rods demonstrated 30% higher drug loading capacity and carboxylation, further increased it to 42%. The in vitro drug release profile was analysed in nasopharyngeal carcinoma cell CNE‐2 cells, with IC50 value at 50 nM, which achieved the lower DOX concentration than that achieved via the clathrin-mediated pathway of internalisation. This shows that the rod-shaped MSN could deliver the loaded drug to tumour cells with better efficiency than the spherical MSN. This contradicts the new theories about the advantages of spherical shape in drug delivery [310]. Concurrently, maintaining and delivering the aspect ratio particles by inhalation route may be challenging [311].

In a similar way, a multi-layer chitosan modified MSN rod hybrid was synthesised for the delivery of docetaxel in lung cancer cells. MSN was synthesised with slight modification in the CTAB mediated synthesis and the MSN rod were further conjugated with chitosan using cross-linking polymerisation. The size of the nanorods was 80 ± 17 nm in width, and 260 ± 25 nm in length and the specific surface area was noted as 945 m^2^g^−1^, with an average pore size of 2.5 nm and a pore volume of 1.13 mLg^−1^. The high surface area and large pore volume of MSN resulted in a 90% drug delivery within 48 h in the A549 cells [245].

**Figure 8.**
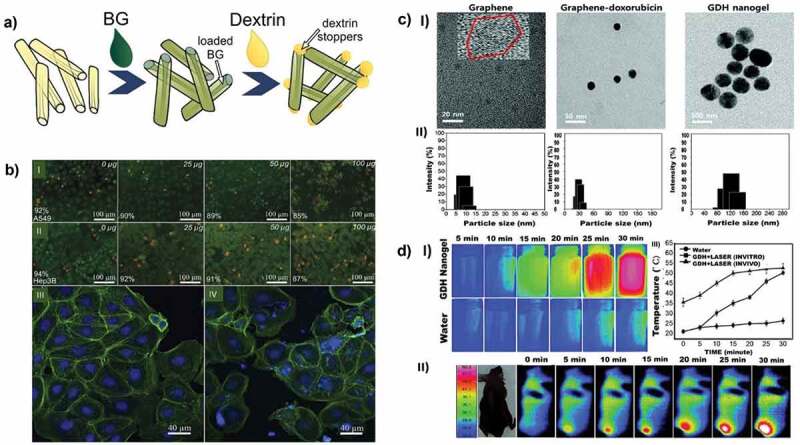
Porous nanoconjugates used for cancer therapy studies. A) Schematic representation of brilliant green-loaded HNTs and the addition of dextrin stoppers. B) Human cells viability with DX- HNTs. Fluorescence microscopy studies of viable A549 (I) and Hep3b (II) cells; F-actin filaments stained in intact (III) and (IV) DX-HNTS- treated A549 cells. C) (I) TEM and HRTEM of graphene, the graphene–DOX and the nanogel. (II) The particle size of graphene, the graphene–DOX and the nanogel. D) In vitro (I) and in vivo (III) photothermal images of the nanogel. (II). The nanogels were injected intravenously into the mice tumor. (Reproduced under the Creative Commons license (2015) CC-BY 8A,8B [313]. Scientific reports, Nature Open Access, 8C,8D [315]. (Copyright 2015) Nanoscale, Royal Society of Chemistry.

In addition to the reports on inorganic and organic nanohybrids, another promising nanomaterial for drug delivery is halloysite nanotube (HNT). Halloysite, is a naturally occurring aluminosilicate clay with unique tubular morphology with 25–100 nm diameter and is considered a promising drug delivery carrier for lung cancers due to its low toxicity [312]. HNT loaded with a model dye triazole brilliant green (Figure 8A) and covered with dextrin was tested as a drug delivery vehicle. The triazole brilliant green is capable to kill mitochondria of the malignant cells. The dextrin molecule seals the pores of the HNT, retaining the drug inside the tube (Figure 8B). Dextrin is cleaved by intercellular glycosol hydrolases opening the pores and thus releasing the drug intracellularly [313]. A high drug loading of 4 mg/mg of HNT was achieved by this method. The delivery efficiency of HNT was analysed in both suspended (Hep3b) and the attached cell lines (A549). It is also noted that the uptake of drug in the A549 cells is higher than the Hep3b, but the dextrin didn’t demonstrate a stopper effect in the A549 cells, and the difference in the drug uptake could be due to the differential uptake of the particles by different cells.

Black phosphorus, a layered semiconductor with wide adsorption in the UV range, has been used to form a sandwiched structure with MSNs for combining phototherapy and chemotherapy. The hybrid nanosheet structure was used to load Dichloro(1,2-Diaminocyclohexane) Platinum(II) (DACHPt) and cisplatin drugs into the MSN. The use of the black phosphorus-silica sandwich structure reduced the lung metastasis by complete photothermal degradation of the system. This functionalization and modification resulted in the high drug loading and the resultant tumour inhibition by combining the chemotherapy and photothermal therapy [314]. In yet another approach, Khatun et al. demonstrated a multifunctional thermo and red light responsive chemotherapeutic nanogel comprising of functionalised graphene and DOX (Figure 8C). The pH-sensitive disulfide bond was used for conjugation with hyaluronic acid to achieve targeting. A high tumour inhibition was achieved in the near-infrared region in both A549 cells and in the in vivo mice models (Figure 8 D I–III). The main drawback of this system is its low tumour penetration capability [315].

**Figure 9.**
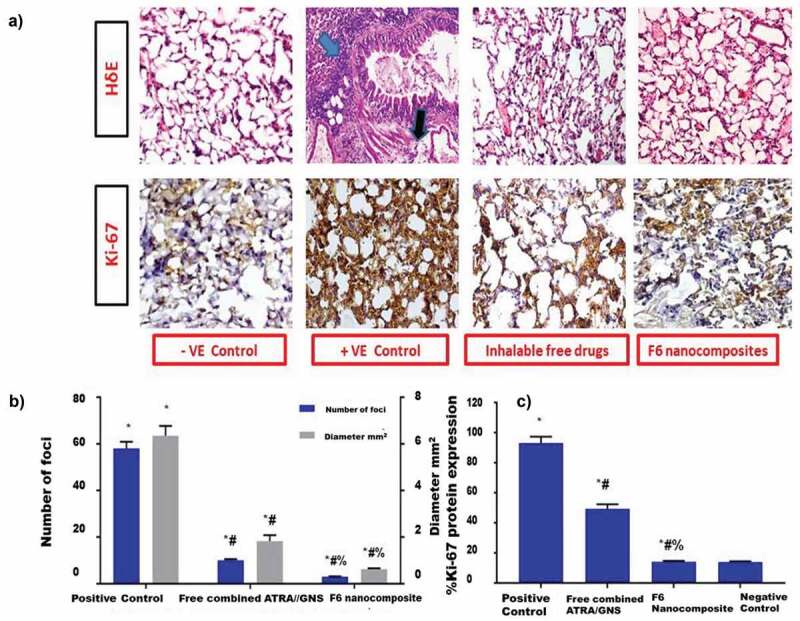
Effect of inhalable dual-targeted hybrid lipid-protein core-shell nanocomposites. (A) Histopathological images of lung sections of different groups and immunohistochemical analysis of Ki-67 collected from lung sections of treated mice; (B) Effect of treatment on the number and the diameter of the lung tumor; (C) Different treatment groups -% Ki-67 proliferation markers. (Reproduced with the Permission 9 [256]. (Copyright 2020) ACS Biomater. Sci. Eng., American Chemical Society).

Several discrete studies have been conducted on combining MSNs and other porous materials to construct hybrid structures for combining drug delivery with other approaches such as targeting, phototherapy, diagnostics and imaging, these approaches are versatile and depict the promise of using such hybrid structures for potential drug delivery applications [202,316,317]. At the same time, the in vivo bio-distribution and safety confirmed with these hybrid nanosystems vary drastically. For example, the in vitro trials confirmed from the A549 cell line are finalised by treating CT26 implanted mice, which is not satisfactory. The study could have been conducted using the A549 tumour model [253]. Another study focused on thermo-responsive DOX release to lung carcinoma cells achieved using porous silica after hybridizing with PNIPAAm (poly-N(iso propyl acrylamide) by radiofrequency enhanced magnetic hyperthermia. The in vivo study on lung carcinoma (MLL) models reveals the high efficacy of such NPs. However, the study could have focused on its potential long-term toxicity as PNIPAAm may be unsuitable for long-term biological applications. Other than these studies, for localized lung cancer therapy, some inhalable dual-targeted hybrid lipid-protein core-shell nanocomposites (HLPNPs) co-loaded with ATRA and GNS were prepared. The spray-dried NPs showed an improved antitumour efficacy (Figure 9) with inhalable nanocomposites prepared using mannitol:HPβCD:leucine in 1.5:1.5:1 mass ratio and showed a deep lung deposition, as demonstrated by their small MMAD (2.47 μm) and high FPF (70.81%). Although nanohybrids have been reported for lung cancer treatment and there is a growing body of evidence for the efficacy of nanohybrid treatments, the lack of research into long-term toxicity is the main limitation. Therefore, future studies should be focused and addressed mainly on these issues [256].

Dendrimers have also been explored as drug carriers in lung cancer treatment. Polyamidoamine (PAMAM) dendrimers have gained attention due to the monodispersed tree like structures with various functional groups on the surface. Liu et al. has functionalised the surface of the PAMAM with lung cancer targeting peptide LCTP; RCPLSHSLICY that can specifically target NSCLC cells. The results showed that the intracellular uptake of the targeted dendrimers in NCI-H460 was higher than the non-targeted dendrimers. The in vivo results in BALB/c-nu/nu athymic mice with lung cancer xenografts also shows a higher internalisation within 4 h of intravenous injection to the tumor site than the non- targeted dendrimers [318] while the tumor reduction efficiency was not evaluated. Dendrimers are even explored for gene therapies which have been used for selective transfection of genes to the lung cancers. In a recent study, a targeting peptide with high binding affinity for lung cancer was attached with polyamidoamine dendrimers. The positively charged dendrimers electrostatically bind to the negatively charged nucleic acids, inhibit endogenous nucleases and transfect cells targeted by the attached peptide [319]. To reduce the dose dependent side effects of the drugs, a chemo and biologic combinatorial drug delivery of the folate receptor functionalised dendrimers has also been explored for the lung cancer treatment. A folic acid (FA)-conjugated polyamidoamine dendrimer (Den)-based nanoparticle (NP) system for co-delivery of siRNA against HuR mRNA (HuR siRNA) and cis-diamine platinum (CDDP) to folate receptor-α (FRA)-overexpressing H1299 lung cancer cells was developed. The co-delivery of HuR siRNA and CDDP using the FRA-targeted NP had a significantly greater therapeutic effect than the individual therapeutics. The results show a significant reduction in the tumor proliferation with negligible toxicity towards the MRC9 lung normal fibroblast cells [320]. Hopefully, more hybrid nanostructures with dendrimers will be succeeding the lung cancer treatment in the future.

## 5. Stimuli-responsive drug delivery materials in lung cancer therapy

**Scheme 7.**
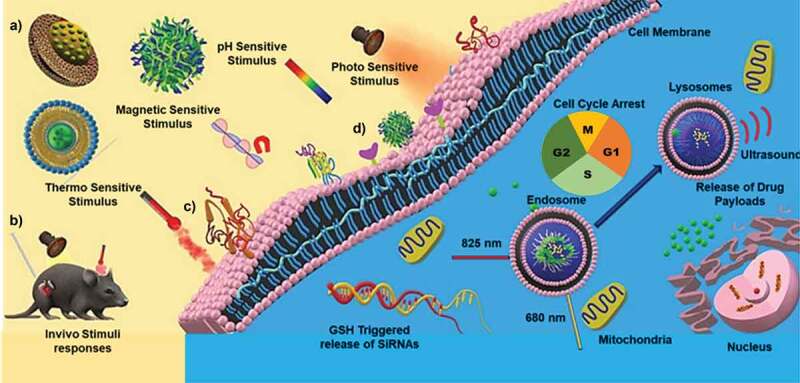
Schematic depiction of stimuli-responsive drug delivery. A) different nanoparticles for drug delivery B) in vivo stimuli-responsive experiments in mouse C) Different Target Receptors for combining targeting with stimuli responsiveness D) Receptor-mediated endocytosis and payload release.

Controlling the release of drugs from drug delivery vehicles is important to avoid leakage and the accumulation of drugs in unwanted areas. While drug targeting can ensure that the cargo is taken to the right tumour location, it does not control the unwanted release of the drug during circulation. The controlled cargo release mechanism (Scheme 7) from the nanoporous materials can be achieved by the attachment (or loading) of drugs using specific linkages (or pore blocking agents) that release the drug from the cargo based on the specific endogenous and exogenous stimulus. The stimuli response can be achieved by attaching various broad-spectrum stimuli-responsive gate-keepers to block the pores of porous nanomaterials and various target receptors [321].

Different gate-keepers can be attached via chemical bonding or through molecular interactions for the controlled release of drugs. These stimuli-responsive gate-keepers undergo hydrolytic cleavage, protonation or a molecular conformational change to detach from the drug delivery carriers for drug release. The concept of stimuli-responsive drug delivery was first explained in 1970 by the development of thermosensitive liposomes [322]. Physiological conditions present within the biological systems such as acidic pH, higher glutathione concentration and release of specific enzymes are considered endogenous stimuli. The use of external factors such as temperature, magnetic field, ultrasound, electric field, and light for drug release is termed as exogenous stimuli. Recently studied stimuli responsive porous drug delivery agents are discussed in the section below.

### 5.1 Endogenous stimuli-responsive drug delivery

## 5.1.1 pH-sensitive responses

Drug delivery as a response to pH is an efficient method for targeted drug delivery. Usually, the surface of the nanoporous materials is modified by pH-sensitive polymeric ligands that are detached or destroyed in an acidic stimuli environment through some conformational change or solubility change while keeping the nanocarriers safe in the normal pH of the blood circulation by stealth effect. Combining porosity and the stimulus on the surface is an efficient way of targeting the tumour site with more accuracy [323,324].

**Figure 10.**
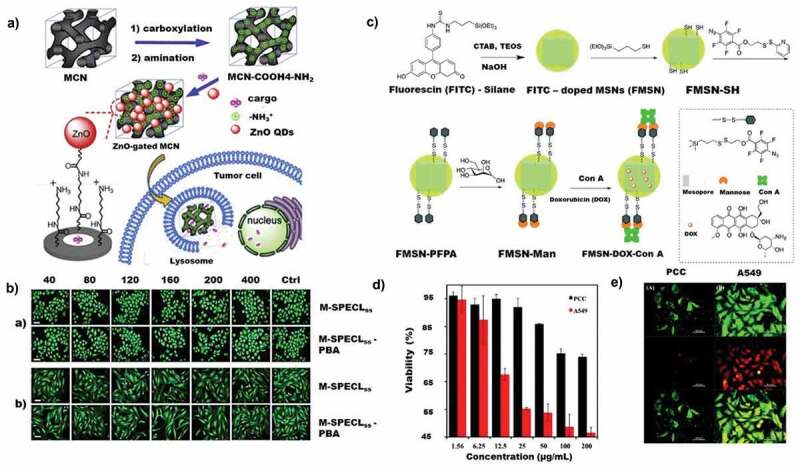
Endogenous stimuli responses and corresponding effect in cancer cells. A) ZnO- gated MCN controlled delivery system and release of encapsulated cargo. B) Live-cell staining by calcein dyeing of (a) HEPG2 cells and (b) OB cells which were incubated with different concentrations of M-SPECL_SS_ and M-SPECL_SS_-PBA showed high cytocompatibility. (Scale bars: 20 μm). C) Schematic representation of the synthesis of Con A-gated, FITC-doped, MS glyconanoparticles. D) Percent viability of A549 and PCC cells against FMSN-DOX-ConA particles. (Reproduced with permission from 10A [325]. (Copyright 2016) Carbon, Elsevier, 10B [340]. (Copyright 2016) Acta Biomaterialia, Elsevier, 10C,10D,10E [342]. (Copyright 2015) Chemical Communications, Royal Society of Chemistry).

In a typical study of pH-responsive gateways, Huang and co-workers have explored the dissolution mechanism of ZnO quantum dots in acidic pH. Mesoporous carbon nanoparticles (MC) were loaded with rhodamine 6 G (Rh6G) through electrostatic interactions and conjugated with ZnO via amide linkage (Figure 10A) [325]. The MCN were synthesised using the modified Stӧber method and exhibited a specific surface area of 1575 m^2^/g with an approximate pore size of 2.2 nm. After the amide functionalization, the specific surface area was reduced to 881 m^2^/g. The Rh6G interacts with the aromatic domains of the MCN through π–π interactions, making a strong bond with MC at a neutral pH. Zinc oxide quantum dots dissolved in the acidic media (pH 5.5), resulting in the release of the drugs into the tumour environment. A 65% toxicity was observed at a dose of 0.066 ng/cell with little toxicity of naïve ZnO-gated MC in A549 cells. The total sedimentation coefficient of these particles and the corresponding toxicity in in vivo system need to be analysed for further validation.

A multifunctional zinc-based hollow nanoplatform as a smart magnetic pH-responsive drug delivery system for cancer therapy using DOX as an anticancer drug was designed. These fluorescent moieties have several properties such as optical, magnetic with pH-sensitive properties and provide a multifunctional platform that depicted a high tumour inhibition of 82% [326]. In a similar way, a pH-sensitive amphiphilic diblock polymer (poly (PDM-b-PEGMA)) has been grafted on the hollow MSNs via atom transfer radical polymerization (ATRP). The polymeric nanoparticles showed a high drug loading efficiency of 80% using DOX and a complete release in acidic media [327]. Similarly, the pH-controlled release was tested using pSi in lung cancer (A549) cells. Näkki et al. developed a magnetic pH-responsive pSi loaded with DOX hybrid, which showed a higher drug loading efficiency with a longer circulation time of 45 days with no systemic toxicity [328].

Functionalisation of the surface by modification with various polymers is another method to give stimuli responsiveness to the nanosystem. Chen et al. modified the surface of MSNs with Poly(ethyl methacrylate) (PPEMA) and poly(ethylene glycol) (PEG), in which PEG was used for achieving a high dispersity. At the neutral pH, PPEMA is hydrophobic and blocked the pores of MSNs, which in turn block the drug release; however, the corresponding drug release was observed in the acidic tumour environment. The promising results show a successful pH mediated controlled release of DOX at acidic pH of 6.5 and 5, which further reduced the premature drug loss with high therapeutic feasibility [329]. The receptor-mediated targeted drug delivery that targets the folic acid receptors of the lung cancer cells with a stimulus responsive amino hyperbranched polymer attached to the carboxyl functionalised surface of the MSNs was used to deliver tetrahedrine. The drug loading of around 27% was achieved by the MSN nanocomposites [330]. Targeting the same folic acid receptors, folic acid conjugated MSN was combined with MRP-1 siRNA, which improves the suppressive effects of the bioactive compound myricetin on non-small cell lung cancer. Both proliferation and cell viability show that the porous silica nanoparticles have strong clinical significance in lung tumour suppression [331].

Lipid coated MSNs have also been used for pH-responsive tumour targeting in lung cancers [332–334]. 1,5 dioctadecyl-L-glutamyl 2-histidyl-hexahydrobenzoic acid (HHG_2_C_18_) was coated over amino functionalised MSN for co-delivering targeting drug erlotinib and DOX to the A549 cells. A Soy phosphatidylcholine (SPC) is used a lipid precursor for lipid film coating and the drug erlotinib was added to the film for the synthesis of SPC-ER that was further combined with DOX loaded amine functionalised MSN through ultrasonic dispersion. The zeta potential of the particles changed from −38 mV to 4.5 mV with a change of pH value from 7.4 to 6.5 and turned 22 mv at pH 5.5 or 4.5 due to the protonation of carboxyl group of hexahydrobenzoic acid and the amino group of histidine [335]. This lipid coating reversed the negative zeta potential of the MSN and led to a sequentially staggered release of both the drugs, which was further confirmed with different pH release studies.

Polydopamine is melanin like adhesion protein that easily dissociates in acidic media. This unique property is used as a tool to deliver the drugs and escape protein corona formation. Moreover, the functional group quinone in the dopamine can react with both the thiol and amino groups by Schiff’s base or Michael addition reactions making its conjugation a simple task. Engineering polyzwitterion and polydopamine decorated on MSNs loaded with the drug DOX have also been examined for pH-sensitive drug delivery. Since these modified particles have been shown to be effective in other cancers, these potential applications can be co-adapted for lung cancer treatments [336].

Other than the MSN-based systems, some nanovesicles were also explored for pH mediated drug delivery. Joshi et al. developed an endogenous lung surfactant mimetic lipid nanovesicle from 1,2-dipalmitoyl-sn-glycero-3-phosphocholine (DPPC). DPPC is an abundant surfactant in the lungs. A Combination of DPCC and 1,2-dioleoyl-sn-glycero-3-phosphoethanolamine (DOPE) was used to form lipid vesicles. These nanovesicles were used to encapsulate chemo drug paclitaxel. The pulmonary surfactant helped to penetrate through upper airways and improved aerosolization and stability of the lipid vesicle. It showed higher cytotoxicity in A549 cells and high cytocompatibility in the normal lung fibroblast cells (MRC5), suggesting that the cytotoxicity is high only in the tumour cells due to the change in the tumour microenvironment. This study is very promising and opens the door for controlling the stability of the particle by lipid encapsulation for inhalation therapy with minimal drawbacks [337].

### 5.1.2 Redox sensitive responses

The redox-sensitive response uses specific redox enzymes such as the glutathione (GSH) to cleave redox-sensitive bonds. The concentration of GSH within the tumour microenvironment is high owing to high ROS production. GSH can selectively cleave the disulphide bonds and thus can be used as a strategic enzyme for stimuli-responsive release [338]. In a particular study, it was shown that the GSH levels in the lung tumours are in the range of 2 to 60 nmol/mg-protein or 1–3 µmol/g-tissue [339]. Recent studies indicated that the change of redox potential is a key molecular mechanism for exerting tumour growth in various cancers and can act as oncological indicators [340]. Although several nanoparticles are reported for redox responsiveness in lung cancers, a few studies related to the porous structures in lung cancer are noted here.

Redox responsive star-shaped magnetic micelles have been developed for efficient drug delivery to lung cancer cells. The micelles were composed of self-assembled poly (ethylene glycol) (PEG)-poly(e-caprolactone) (PCL) copolymer linked with disulphide bonds. The hydrophobic core of the micelle is loaded with DOX and magnetic iron oxide particles were adsorbed through physisorption. Finally, these molecules were coated further with the PEG for easy internalisation and targeting [341]. A high cytocompatibility of the non-targeted (M-SPECLSS) and targeted magnetic star-shaped polymer micelles (M-SPECLSS-PBA) in HepG2 cells and OB cells has been observed using Calcein dyeing (Figure 10B). The investigation on the redox response showed that at 10 mM GSH, the signal of the PEG became weaker which lasted for a maximum of 24 hrs and without GSH, the nanohybrid structure was intact. Even low DOX concentration, loading of 6.2% was observed, demonstrating a dose-dependent growth inhibitory effect that was about 1.4 fold higher than the normal DOX. The high efficacy comes from the fact that the polymeric micelle can be selectively cleaved by GSH within the tumour microenvironment, releasing a large amount of drug selectively within the tumour cells.

Redox responsiveness can be initiated through several other gated channels. Zhou et al. used the protein-carbohydrate recognition as a gating factor for redox responses and the corresponding drug release from MSN. The study utilised the lectin gated mesoporous glyconanoparticles. Lectins are non-immunogenic proteins that can interact with carbohydrates. The fluorescent MSN were functionalised with D-mannose using a photochemical nitrene-mediated approach and then modified again with redox-sensitive disulphide linkers (Figure 10C). These linkers were further attached with the per fluorophenyl azide (PFPA) groups for the glutathione responsive drug release [342]. The cell internalization studies and the release profile of the DOX from the gated MSN in lung cancer cells have been confirmed using further FITC conjugation (Figure 10D, 10E). The high glutathione concentration in the tumours destroyed the lectin gatekeepers and released the drug to the environment, resulting in 61% of drug release in 7 hours. These recent studies demonstrated a trend towards developing biological components and their hybrids for drug delivery due to the reduction in side effects.

Other than MSN based system, a hyaluronic acid-vitamin E succinate conjugate has also been reported for the redox-sensitive drug release of PTX in the lung cancer cells. Vitamin E succinate (VES) is a derivative of tocopherol and has been utilised in several cancer studies and showed higher drug loading efficiency. A VES based redox-sensitive nanocarrier with hyaluronic acid and a core-shell structure has been proposed. The overall size of the nanosystem was 200 nm, with a loading capacity of 33% and an entrapment efficiency of 90%. The disulfide modification on the surface of the VES breaks at higher GSH concentration releasing the drug and resulting in 70% apoptosis in cancer cells that was higher than the individual drug, drug+ VES and drug + HA [343].

### 5.1.3 Enzyme sensitive responses

Enzyme sensitive functional nanoporous materials have been explored for lung cancer therapy as well. In fact, matrix-metalloproteinases (MMPs) are overexpressed in many types of cancer. This targeted approach can be applied for obtaining enhanced bioavailability through guided therapeutic targeting. To realize this strategy, MMP responsive NPs based on a functionalised copolymer mPEG-Peptide-PCL was used along with curcumin drug and were well internalized into cancer cells through macropinocytosis. The pharmacodynamics and cellular internalization analysis in vitro, and in vivo biodistribution studies confirmed that these enzyme responsive curcumin loaded nanoparticles showed better targetability and thus improved therapeutic outcome than the intact curcumin in the tumour tissue [344].

Another study by Yanmei et al. showed that a cascade targeting, multi drug-loaded, core-shell nanoparticles (DLTPT) consisting of CD44 specific hyaluronic acid shells decorated with DOX (D), whereas the core containing lonidamine (L) loaded triphenylphosphonium (TP) mitochondria targeting (T) nanoparticle to finally form ~ DLTPT. The as-synthesised DLTPT had prolonged blood circulation time and efficient tumour accumulation due to their selective mode of action. When applied, the HA-DOX shell can be disintegrated by extracellular hyaluronidase enzymes, changing the overall particle size and charge reversal from negative to positive, increasing the tumour penetration and thereby enhanced cellular internalization. The fast degradation of HA-DOX further enhanced the DOX release, whereas the exposed cationic LTPT core can undergo a rapid endosomal escape and thereby targeting to mitochondria for ionidamine delivery. It should be noted that when DLTPT was combined with immunotherapeutic agent like anti-PD-L1 (programmed death ligand-1), the tumour growth was inhibited significantly, inducing immune responses against lung metastatic tumour model in vivo [345].

### 5.2 Exogenous stimuli-responsive drug delivery

#### 5.2.1 Temperature-sensitive response

Temperature-induced drug delivery utilizes a temperature-induced conformational change of the nanostructure or the dissociation of the attached surface ligands through a sharp increase in temperature. This stimulus is used for the rapid disassociation/decoupling of the drugs at specific temperatures (∼ 40–42°C). The characteristic feature of the thermo responsive drug delivery carrier is that it must be stable at temperatures above 37°C.

**Figure 11.**
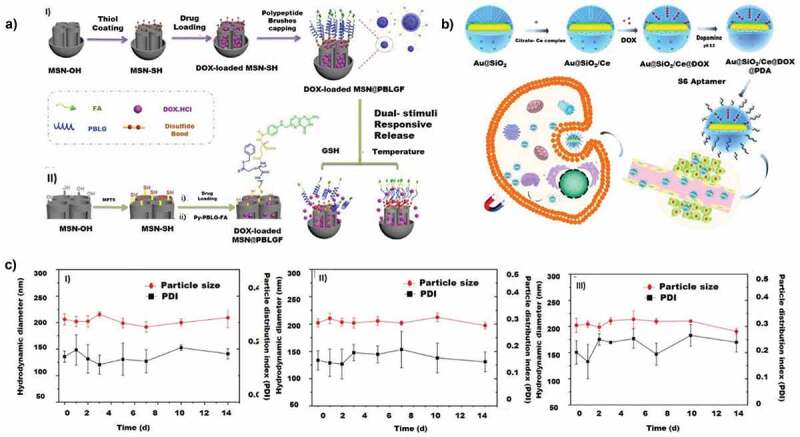
Exogenous stimuli responses and corresponding effect in cancer cells. A) Schematic illustration of MSN@PBLGF nanoplatform and its operation in response to GSH and temperature dual-stimuli for targeted drug delivery. B) Schematic representation of Au NR@SiO_2_/Ce@Dox@PDA @aptamer magnetic nanocomposites. C) Studies on the stability of Au@SiO_2_/Ce@PDA@aptamer nanocomposites in (I) PBS, (II) serum and (III) 5X diluted PBS . (Reproduced with the permission from 11A [347]. (Copyright 2019) Chinese Chemical Letters, Elsevier, 11B, 11C [351]. (Copyright 2019) New Journal of Chemistry, Royal Society of Chemistry).

Cho et al. coated the MSN with multiblock copolymer – poly(ethylene glycol)/poly(epsilon caprolactone) (PEG/PCL) as gatekeepers. On the introduction of heat stimuli, the PCL crystals melt, opening the matrix for the release of the drug (DOX). In the absence of the heat shock, only 0.2 mg of DOX was released out of the 9.4 mg of the loaded drug; however, in the presence of a heat shock, a sustained release of the complete drug was achieved within 12 hrs. The heat-induced damage to normal cells near the tumour cells is a potential drawback of such a system [346]. A temperature-sensitive polypeptide brush, made from hydrophobic poly(γ-benzyl-l-glutamate) (PBLG) (Figure 11A), coated MSN with folate receptors for targeting cancer cells has been reported for dual responsive drug release. Both the GSH dependent release and the temperature-dependent release has been conducted. Still, compared to the PEG/PCL polymer described above, these PBLG modified MSN released the drug within 6 hrs even though the drug loading efficiency is almost the same [347]. A significant change has been observed in hypoxia inducible factor (HIF-2α) expression and activity in lung cancer cells subjected to hyperthermia-induced drug delivery. Further research on the related perspectives will shed light on better hyperthermia-induced drug delivery and treatment [341].

#### 5.2.2 Photodynamic responses and magnetic triggered responses

The photodynamic response is activated by light of a specific wavelength and produce reactive oxygen species causing site-specific cell death [348]. A photoresponsive agent is activated to destroy cancer cells or initiate a drug release with the illumination of the light of a specific wavelength. There are very few reports on the individual responsive systems, and the current trend is more focused on combining photo and magnetic responsive therapies [349]. Biodegradable albumin and biocompatible and ultra-small hybrid of copper sulfide nanosheets (CuS@BSA) has been fabricated on the surface of hollow MSNs for PTX delivery to the lungs. Firstly, the CuS@BSA has been synthesised using one step biomineralization, and this was attached to the surface of the DOX loaded MSN via disulfide conjugation. The hybrid successfully generated thermal responsive delivery of the chemo drug and combined the photothermal therapy with imaging in a single platform. The photothermal efficiency was 51.5% using an 808 nm laser and different power densities (0.5–1.2 W cm^−2^). These biocompatible hybrid conjugated MSNs degrade in the system and deliver the drug due to induced hyperthermia with negligible toxicity. The data suggested that the fabricated platform resulted in <20% cell survival at lower concentration of DOX (5 µg/mL) [350].

In another study, a citrate-Ce complex hybridised with gold nanorods coated with MSN was developed for the dual responsiveness to the A549 lung cancer cells with high tumour inhibition (schematically represented in Figure 11B). This nanoparticulate system combines four different responses – targeting (from an aptamer conjugation), near-infrared photothermal response (from gold), pH response (from polydopamine as pore blockers) and magnetic response (from Ce-complex) to achieve a multifunctional drug delivery and therapy system. The maximum drug loading achieved using this system was only 23%, which is less than other systems used for loading DOX. The nanoparticles could be accumulated at a tumour site by applying a magnetic field due to the high magnetic activity of the Ce-citrate complex. In an acidic tumour environment, the polydopamine dissolves, releasing the drug molecules. The gold nanorod nanocomposite is highly stable (Figure 11C) and produced localised hyperthermia in near infrared light promoting drug release and localised cell damage. A 3.3 times higher DOX release was observed at an acidic pH of 5.0 as compared to the neutral pH confirming the pH sensitivity of the system. The cell viability of this composite was 33.8% at 10 µM DOX concentration which is consistent with other carriers [351].

## 6. Summary and future directions

There is no doubt that the nanoporous materials with tunable porous structure, morphology, specific surface functionalization and different geometries are powerful supports for the delivery of drugs for the cancer treatment. These nanoporous materials can be readily synthesised thanks to the availability of different fabrication process. In this review, we have briefly summarised the role of porous drug delivery materials in the treatment of lung cancer. There is substantial diversity in the types of suitable materials for lung cancer therapy owing to an additional inhalation mode of drug delivery, in addition to the systemic and oral delivery. As described in this review, the inhalation therapy requires specific physico-chemical parameters that are favourable for porous materials-based drug delivery due to their unique aerodynamic properties and low density. Several organic, inorganic, bio-based materials and their nanohybrids have been well explored for drug delivery applications in lung cancer. Among different types of materials used as DDS, silica-based nanomaterials and polymer/lipids have been researched extensively as DDS for the treatment of lung cancer however, limited studies have been conducted on the layered 2D inorganic based hybrid nanomaterials. The size, shape and the surface charge of nanoporous materials have different effects on systemic and inhalation modes of drug administration. Small sized particles (less than 200 nm) and the spherical morphology are crucial factors in oral drug delivery and other intravenous administartion. However, the aerodynamics of inhalation route of administration demands a larger size (300 nm −500 nm) for drug delivery which is an additional advantage for the porous materials in lung cancer treatment. Further research on the effect of size, shape and surface charge of the porous particles will pave the way for development of novel DDS in the lung cancer treatment.

It should be noted that the requirement of these nanoporous materials for the effective drug delivery for the lung cancer treatment is so complex that the pure nanoporous materials without proper functionalization cannot satisfy the need for the best drug delivery system. The unique surface properties together with the functional groups on the surface and their versatility in providing different surface platforms offer new opportunities for the efficient drug delivery. For example, the proper functionalization with the targeting molecules or other hybrids system can be one of the solutions to overcome the problem of site-specific delivery of the drugs. Therefore, the focus of the current research seems to be shaping towards the use of combination of different materials for combining different forms of treatment and controlling the release of the drugs. As can be explained in this review, stimuli responsive drug delivery using hybrid materials is a convenient method to control the release of the drugs and prevent its side effects. Among various hybrids, blending mesoporous silica-based NPs with active targeting moieties to improve the tumour tissue selectivity of drug loaded porous nanomaterials is a good strategy for increasing its efficacy. Photo therapeutics, such as, organic dyes like IR-780 or ICG can also be combined along with hybridized porous organic/inorganic/bio structures to get an improved photo-thermal as well as photo-dynamic therapeutic outcomes. One of the advantages of combining different materials for drug delivery is that it can offer unique opportunity for the development of theranostic materials for the treatment of lung cancer. Even though there have been tremendous improvements in theranostic approaches using wide variety of functional porous nanomaterials, it was noted that the major limitation associated with the theranostic approaches is the lack of pre-clinical assessments.

While hybridisation of nanomaterials seems to be the main theme going forward in the research on drug delivery materials for lung cancer, it is unclear whether such an approach is commercially feasible. Owing to the stringent requirements of regulatory authorities, the synthesis and use of nanomaterials as drug delivery agent has been very challenging with very few approvals of nanomedicine. Majority of the approvals thus far have been based on liposomal or polymeric drug delivery agents with a few exception of iron oxide based nanomaterials as nanomedicine for diagnostic applications. Considering these facts, it is hard to envisage the commercial applicability of nanohybrids given their complicated procedures. The researchers working in these areas should give due importance to the scalability of the synthesis approaches as well as place an emphasis on reducing the variability in different physico-chemical properties such as size, shape, surface charge and heterogeneity in chemistry. Perhaps, this explains the limited availability of clinically relevant research data on the use of hybrid nanomaterials and presents an opportunity for new research in this area.

Despite the limitations associated with reported research related to the drug delivery applications of porous materials in the treatment of lung cancer, one has to appreciate the synergistic merger of the nano-chemistry and nano-technology achieved to improve the targeting functions and drug efficacies in most of the reported nanohybrids. However, there are many unanswered questions due to the fact that many studies were limited only at the in vitro level without a detailed and thorough analyses for nuclear level toxicology of such emerging porous nanomaterials. Another area that require a lot of attention is the structure–property relationship of these unique nanostructures and the theoretical calculations for understanding the interaction between these nanoporous hybrid drugs and the macrophages, cell organelles, and various species and we surmise that this will attract much interest in the future as they will play major role in understanding the effectiveness of these novel drug delivery systems for lung cancer treatment.

In summary, the present review gives an overview of the recent trends in the lung cancer therapy with emerging nanomaterials including organic, inorganic, bio, and their hybrids. We observe that it is extremely important to have a clear understanding on basic toxicology and applicability of these trending porous nanomaterials, which would further enhance the authenticity of these relevant and emerging porous materials to be marketed as an ideal lung cancer medicine in the near future.
